# Interplay of fibroblasts with anaplastic tumor cells promotes follicular thyroid cancer progression

**DOI:** 10.1038/s41598-019-44361-6

**Published:** 2019-05-29

**Authors:** Laura Fozzatti, Vanina Alejandra Alamino, Sunmi Park, Lucila Giusiano, Ximena Volpini, Li Zhao, Cinthia Carolina Stempin, Ana Carolina Donadio, Sheue-yann Cheng, Claudia Gabriela Pellizas

**Affiliations:** 1 Centro de Investigaciones en Bioquímica Clínica e Inmunología - Consejo Nacional de Investigaciones Científicas y Técnicas. Departamento de Bioquímica Clínica, Facultad de Ciencias Químicas, Universidad Nacional de Córdoba, Córdoba, Argentina; 2 Laboratory of Molecular Biology, Center for Cancer Research, National Cancer Institute, National Institutes of Health, Bethesda, Maryland USA

**Keywords:** Cancer metabolism, Cancer microenvironment

## Abstract

Thyroid cancer is the most common endocrine malignancy. Anaplastic thyroid cancer is one of the most aggressive thyroid tumors. It is known that activation of oncogenes and/or inactivation of tumor suppressor genes in tumor cells promotes tumorigenesis. The microenvironment of the tumor also plays a key role on cancer development and progression in a variety of tumors. However, the mechanisms by which tumor-stroma crosstalk in thyroid cancer remains poorly characterized. In this study we aimed to understand how interactions between fibroblasts and anaplastic thyroid cancer cells contribute to thyroid carcinogenesis. We first characterized the phenotypic changes of human fibroblasts *in vitro* through co-cultures by using transwells as well as by using anaplastic thyroid cancer cells-derived conditioned media. We found that fibroblasts acquired an activated phenotype or also known as cancer-associated fibroblast phenotype after being in contact with soluble factors secreted from anaplastic thyroid cancer cells, compared to the fibroblasts in mono-cultures. All the changes were partly mediated through Src/Akt activation. Treatment with the antioxidant N-acetyl-cysteine reversed in part the metabolic phenotype of activated fibroblasts. Remarkably, conditioned media obtained from these activated fibroblasts promoted cell proliferation and invasion of follicular thyroid cancer cell line, FTC-133 cells. Thus, a reciprocal and dynamic interaction exists between tumor and stromal cells, which results in the promotion of thyroid tumorigenesis. The present studies have advanced the understanding of the molecular basis of tumor-stroma communications, enabling identification and targeting of tumor-supportive mechanisms for novel treatment modalities.

## Introduction

Thyroid cancer (TC) is the most common form of endocrine malignancy and their incidence has been increasing rapidly in the past three decades worldwide, mainly in women^1,2^. About 40,000 cases of female TC are expected to be diagnosed in 2018 in the United States^2^. Among the different types of TC, anaplastic thyroid cancer (ATC) is a rare type of cancer but it is one of the most aggressive and chemotherapy-resistant types of all thyroid carcinomas. Characterized by its undifferentiated cells, it spreads quickly to distant organs, mainly lung and bone, and does not respond well to standardized therapy^3,4^. Currently, no effective target treatments are available that can improve overall survival^4,5^. Therefore, there is a critical need to identify new biological targets that can be translated into therapeutic approaches.

The effects of oncogenic transformation of epithelial tumor cells on cancer progression have been well established. More recent evidences, however, have shown that tumor microenvironment (TME) also influences cancer cell behavior and disease progression in many different types of neoplasia^6^. This TME is an active and dynamic component supporting tumor growth, helping the tumor epithelial cell in its survival. The TME comprises, in addition to extracellular matrix, the epithelial cells (tumor cells) and a variety of non-epithelial cells (or stromal cells), being activated fibroblasts one of their main cell components^7^.

Fibroblasts are quiescent in normal conditions. To acquire tumor-supporting phenotypes, normal fibroblasts undergo activation through different mechanisms. Numerous studies have demonstrated that oncogenic paracrine signaling produced by epithelial tumor cells, including growth factors, chemokines and exosomes among others, may recruit fibroblasts to the tumor site and activate quiescent fibroblasts^8–12^. Once activated, fibroblasts [also named myofibroblasts or cancer-associated fibroblasts (CAFs)] become metabolically active, proliferate and secrete soluble factors influencing cancer cell behavior and playing key roles in the process of cancer development and progression in a variety of tumors^7,8,13^.

Despite the availability of numerous reports describing the essential contribution of activated fibroblasts or CAFs on carcinogenesis in many different types of cancer, a detailed understanding of the biology of tumor-stroma crosstalk in thyroid cancer is largely under investigated. Therefore, in order to improve our knowledge of the basis of reciprocal cell-cell interactions in ATC and their influence on tumor progression, in our study we established *in vitro* mono and co-cultures of human fibroblasts and human ATC cells, 8505c and KTC-2. We first investigated the effects of the ATC cells secreted factors on fibroblasts phenotype, to recapitulate the tumor cell secretome effects exerted in the immediate proximity of stromal cells. We also explored the impact of paracrine signals released from fibroblasts after treatment with ATC cells-derived conditioned media (CM), on thyroid tumorigenesis. We found that factors secreted from tumor cells may reprogram the metabolism, phenotype and secretome of fibroblasts acquiring activation features. In parallel, these activated fibroblasts secrete soluble factors to modulate tumor epithelial cell phenotype, including cell proliferation and invasion of FTC-133 cells, potentiating thyroid cancer progression. Based on these observations, our results suggest the presence of a paracrine loop between tumor cells and stromal fibroblasts in TC which ends in the promotion of TC aggressiveness.

## Results

### Metabolic and phenotypic reprogramming of human fibroblasts induced by interactions between tumor and stromal cells in co-cultures

**Figure 1:**
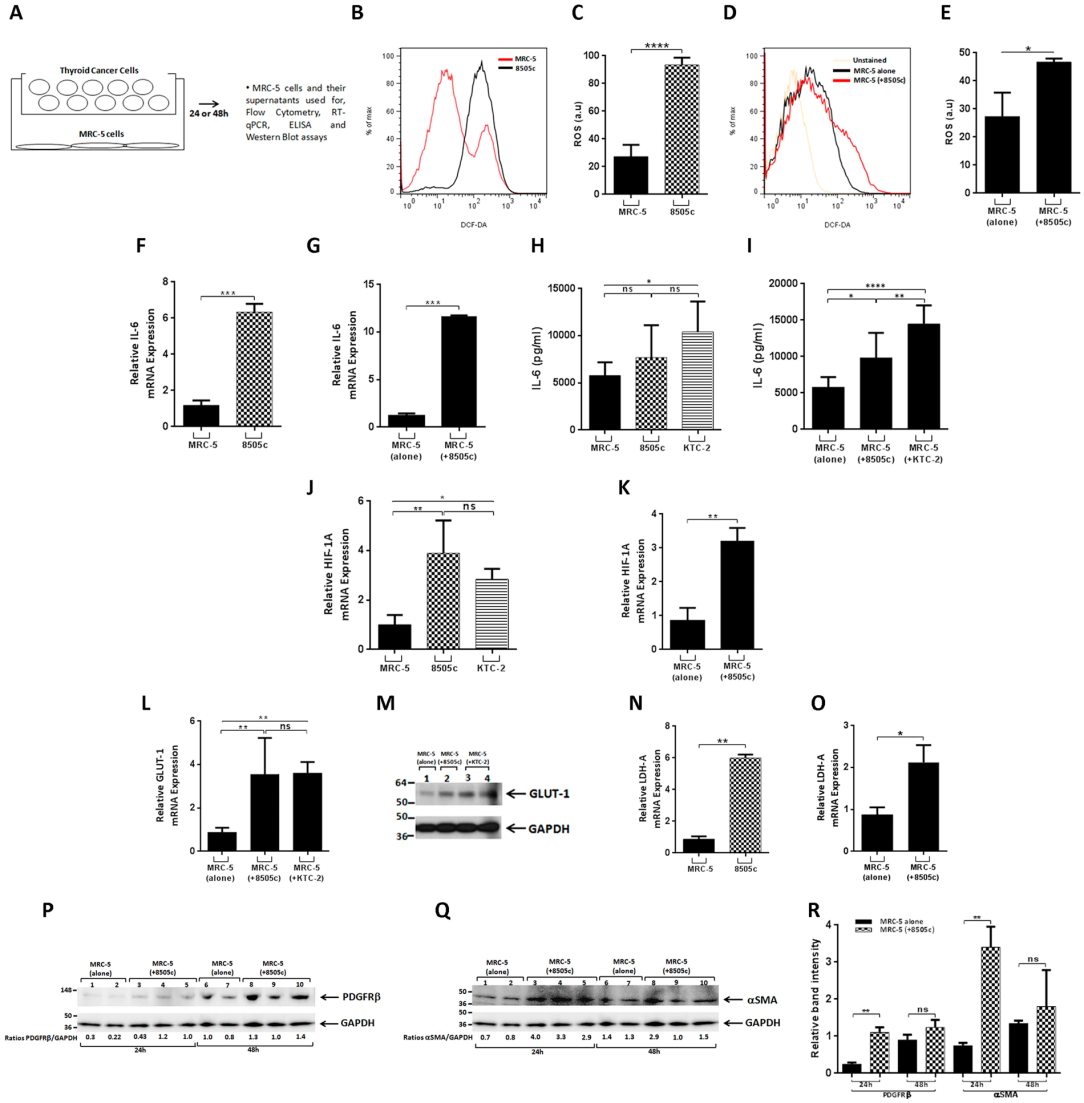
Co-cultures of ATC cells with fibroblasts modify the MRC-5 cells phenotype. (**A**) Schematic representation of co-cultures by using transwells. Total intracellular levels of ROS in MRC-5 and 8505c cells. (**B**–**E**) Basal ROS production in mono-cultures of MRC-5 and 8505c cells: representative histogram (**B**), and quantification (**C**). ROS production in MRC-5 after 48 h of co-cultures with 8505c: representative histogram (**D**), and quantification (**E**). Data are expressed as mean ± SD of 4 independent experiments (n = 4) with triplicate samples for each experimental group. Expression levels of IL-6 (**F**,**G**). mRNA levels by RT-qPCR in MRC-5 and 8505c mono-cultures (**F**); mRNA in MRC-5 cells after co-culture with 8505c cells for 24 h (**G**). Data are expressed as mean ± SD of 3 independent experiments (n = 3) with triplicate samples for each experimental group. Secreted protein in mono-cultures of fibroblasts and ATC cells by ELISA (**H**); secreted protein in MRC-5 cells after co-culture with ATC cells for 48 h (**I**). Data are expressed as mean ± SD of 4 independent experiments (n = 4) with triplicate samples for each experimental group. Expression levels of HIF-1A (**J**,**K**). mRNA levels by RT-PCR in MRC-5 and ATC cells mono-cultures (**J**) and in MRC-5 cells after co-cultures with 8505c cells (**K**). Data are expressed as mean ± SD of 3 independent experiments (n = 3) with triplicate samples for each experimental group. GLUT-1 expression in MRC-5 cells after co-culture with ATC cells (**L**,**M**). mRNA by RT-PCR in MRC-5 and MRC-5 cells co-cultured with 8505c and KTC-2 cells for 24 h (**L**). Data are expressed as mean ± SD of 3 independent experiments (n = 3) with triplicate samples for each experimental group. Immunoblot analysis in MRC-5 cells after 48 h of co-cultures (**M**). The figure shows a representative Western Blot of 3 independent experiments (n = 3) with duplicate samples for each experimental group. The same blot was stripped and re-blotted using anti-GAPDH antibody for loading control [(**M**); full blot is shown in the Supplemental Information, Full Original Blots-I]. Expression levels of LDH-A (**N**,**O**). mRNA RT-PCR in MRC-5 and 8505c mono-cultures (**N**) and in MRC-5 cells after co-cultures with 8505c cells for 24 h (**O**). Data are expressed as mean ± SD of 3 independent experiments (n = 3) with triplicate samples for each experimental group. Immunoblot analysis of PDGFR-β (**P**) and α-SMA (**Q**) in mono-cultures of MRC-5 cells or after co-cultures with 8505c for 24 h or 48 h. The figure shows representative Western Blots of 3 independent experiments (n = 3) with duplicate (MRC-5 alone) or triplicate (MRC-5 + ATC cell) samples for each experimental group. The same blot was cut or stripped and blotted using anti-GAPDH antibody (Full blot is shown in the Supplemental Information, Full Original Blots-I). Quantification of relative expression of PDGFR-β and α-SMA proteins in human fibroblasts after using GAPDH as loading control (**R**). Data are expressed as mean ± SD. *p < 0.05, **p < 0.005, ***p < 0.0005 and ****p < 0.0001. ns, not significant.

It is well known that the crosstalk between cancer and stromal cells has an essential influence on cancer initiation, development and progression in many tumor types^6,14,15^. However, a detailed comprehension of the basis of these interactions on thyroid tumor progression has not yet been extensively investigated. In order to better understand this interplay in ATC, we first characterized phenotypic changes due to tumor-stromal cells interactions, by co-culturing of human fibroblasts, a key component of the tumor stroma, with ATC cells, in transwell chambers (Fig. 1A). Two different ATC cells, 8505c and KTC-2, were co-cultured with normal lung fibroblasts (MRC-5 cells) for 24 h or 48 h and a variety of parameters were evaluated.

Studies have shown that reactive oxygen species (ROS) can induce the activation of multiple signaling cascades that mediate the oncogenic phenotype^8,16,17^. Figures 1B–E show ROS production in co-cultures of ATC and MRC-5 cells. It has been reported that tumor epithelial cells produce high levels of ROS^16,17^. As expected, we found that mono-cultures of 8505c cells showed a significant higher basal ROS production compared to normal fibroblasts [(Fig. 1B and 1C (quantitative analysis)]. Notably, after 2 days of co-culture with 8505c cells, we observed that MRC-5 cells [labeled as MRC-5 (+8505c)] show a significant increase in ROS production (shown by the shift to the right in Fig. 1D and quantification in Fig. 1E). In contrast, human ATC cells did not show differences in ROS production, under the same culture times and conditions (data not shown).

It has previously been reported that ROS production drives the onset of inflammation in the TME^16^. Besides, previous studies describe that one of the main mechanism of communication between fibroblast and tumor cell is mediated by pro-inflammatory cytokines^8^. We decided to focus our attention on interleukin-6 (IL-6), an acknowledged marker of inflammation and one of the major cytokine in the TME^18^. Furthermore, several reports indicate that this cytokine contributes to tumor progression. We analyzed changes in this inflammatory marker, in co-cultures. Figure 1F shows that 8505c cells expressed higher levels of IL-6 mRNA than MRC-5 cells. Importantly, co-culturing for 24 h with 8505c cells, the expression of IL-6 mRNA in MRC-5 cells was significantly increased by ~10-fold (Fig. 1G). We further determined the secreted IL-6 in the media by enzyme-linked immunosorbent assay (ELISA). IL-6 was previously detected in culture medium of KTC-2 cells^19^. In agreement with those observations, the secreted IL-6 was slightly higher in mono-cultures of KTC cells than MRC-5 cells (Fig. 1H). Most importantly, co-culturing with 8505c [MRC-5 (+8505c)] or KTC-2 cells [MRC-5 (+KTC-2)] for 48 h, the IL-6 secreted by MRC-5 was significantly elevated (Fig. 1I).

Besides inflammation, oxidative stress can activate the hypoxia inducible transcription factor-1α (HIF-1α) in the tumor stroma^20^. We therefore measured the expression levels of this critical transcription factor. High expression levels of HIF-1α have been found in dedifferentiated ATC^21,22^. Consistently, RT-PCR analysis showed significant higher levels in the transcript of HIF-1α in the two ATC cell lines used, than in normal fibroblasts (Fig. 1J). More interestingly, MRC-5 cells increased the expression levels of this transcription factor during co-culturing with 8505c cells (Fig. 1K). Previous reports have shown that HIF-1α induces the expression of glucose transporters and glycolytic enzymes^20,22,23^. Among different subtypes of glucose transporters, glucose transporter 1 (GLUT-1) plays a critical role in tumor progression^22^ and that overexpression of GLUT-1 was reported in thyroid cancer^24^. Consistently, we found that the expression of GLUT-1 in MRC-5 cells during co-culturing with ATC cells was increased at the mRNA levels (Fig. 1L) as well as at the protein levels (Fig. 1M). In agreement with the changes in the levels of GLUT-1, during co-culturing with 8505c cells, the mRNA levels of one of the better characterized glycolytic enzyme, lactate dehydrogenase A (LDH-A)^23^, were also significantly increased by ~2.5-fold in MRC-5 cells [Fig. 1N (mono-cultures) and 1O (co-cultures)].

Numerous reports showed that ROS as well as IL-6 induce the activation of fibroblasts^8,16,17^. To test the possibility that MRC-5 could be activated when co-culturing with ATC, we analyzed the expression levels of two well-known markers of cancer-associated fibroblasts (CAF) phenotype or activated fibroblasts: platelet-derived growth factor receptor–β (PDGFR-β) and α-smooth muscle actin (α-SMA)^7,13^. We found a significantly increased protein levels of PDGFR-β by ~3-fold after co-culturing with 8505c cells for 24 h [(Fig. 1P, compare lanes 1 and 2 (mono-cultures) with lanes 3–5 (co-cultures), and quantitative analysis, Fig. 1R)]. A similar increase was also detected for α-SMA by ~4-fold after co-culturing with 8505c cells for 24 h [(Fig. 1Q, compare lanes 1 and 2 (mono-cultures) with lanes 3–5 (co-cultures), and quantitative analysis, Fig. 1R)]. Taken together, our results strongly indicate that tumor-stromal cells interactions modified fibroblasts phenotype by reprogramming their metabolism; their secretory functions and their distinctive expression patterns of proteins.

### Induction of CAF-markers protein expression in MRC-5 by 8505c- and KTC-2 cells-derived conditioned media (CM)

**Figure 2:**
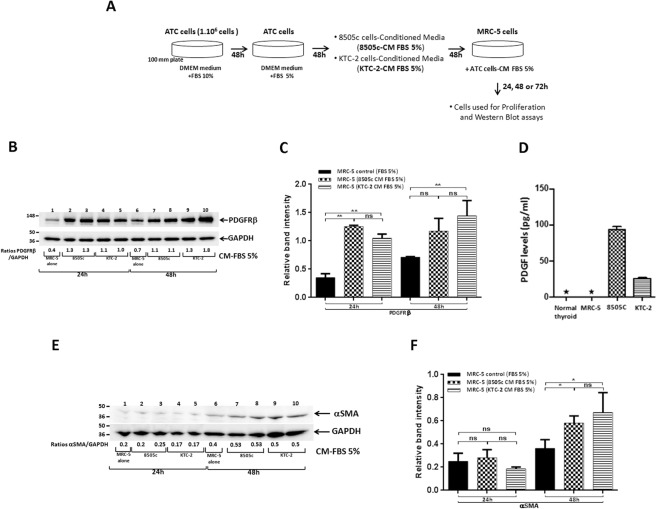
ATC cells-derived CM induced the protein expression of CAF-markers in human fibroblasts. (**A**) Schematic representation of CM preparation from ATC cells and treatment of MRC-5 cells. (**B**–**F**) Effects of CM from ATC cells on CAFs markers in MRC-5 cells. Immunoblot analysis of PDGFR-β in MRC-5 cells grown under normal conditions (control) or exposed to CM derived from ATC cells, 8505c and KTC-2, for 24 h and 48 h (**B**). The figure shows a representative Western Blot of 3 independent experiments (n = 3) with duplicate samples for each experimental group. Quantification of relative expression of PDGFR-β protein in human fibroblasts after using GAPDH as loading control. The blot was cut and blotted using anti-GAPDH antibody. [(**C**) full blot is shown in the Supplemental Information, Full Original Blots-II]. Data are expressed as mean ± SD. (**D**) Concentrations of platelet derived growth factors in the CM of normal thyroid, MRC-5, 8505c and KTC-2 cells determined by ELISA as described in Methods and Materials. Data are expressed as mean ± SD of 2 independent experiments (n = 2) with triplicate samples for each experimental group; *Not detectable. (**E**) Immunoblot analysis of α-SMA in MRC-5 cells grown under normal conditions (control) or exposed to CM derived from ATC cells, 8505c and KTC-2, for 24 h and 48 h. The figure shows a representative Western Blot of 3 independent experiments (n = 3) with duplicate samples for each experimental group. (**F**) Quantification of relative expression of α-SMA protein in human fibroblasts after using GAPDH as loading control. The blot was stripped and re-blotted using anti-GAPDH antibody (full blot is shown in the Supplemental Information, Full Original Blots-II). Data are expressed as mean ± SD. *p < 0.05 and **p < 0.005. ns, not significant.

Our findings by using transwell system (Fig. 1) led us to hypothesize that factors secreted by ATC cells could induce phenotypic changes in MRC-5 cells. To test if transformed thyroid epithelia may activate fibroblasts in a paracrine way, we first investigated the direct effect of factors secreted by ATC cells in the CM on the expression levels of known markers of fibroblast activation as PDGFR-β and α-SMA in MRC-5 cells. In accordance with the transwell system, Western Blot analysis revealed increased abundance of PDGFR-β by ~3- and 1.5-fold in MRC-5 cells incubated for 24 h or 48 h respectively with CM derivate from 8505c cells compared with complete media [Fig. 2B, compare lanes 1 (24 h) and 6 (48 h) (controls, with 5% FBS) with lanes 2 and 3 (24 h) and 7 and 8 (48 h)] and KTC-2-derived CM [Fig. 2B, compare lanes 1 (24 h) and 6 (48 h) (controls, with 5% FBS) with lanes 4 and 5 (24 h) and 9 and 10 (48 h)]. Figure 2C shows the quantitative comparison of the band intensities from Fig. 2B. That PDGFR-β protein levels were increased in MRC-5 cells prompted to us to ascertain whether ATC cells secreted platelet derived growth factors (PDGF) into the media. We found that 8505c and KTC-2 cells secreted high amounts of PDGF (94.5 pg/mL and 26.1 pg/mL, respectively) into the media as compared with normal thyroid cells and MRC-5 cells. PDGF were not detectable in the media derived from normal thyroid cells and MRC-5 cells under the same experimental conditions (Fig. 2D). Thus PDGF could serve as a causative molecule to activate fibroblasts via the elevated PDGFR-β.

Further, we found that another activator marker, α-SMA, was also elevated in MRC-5 [~1.2-fold at 48 h under the same culture conditions; Fig. 2E compare lane 6 (control, with 5% FBS) with lanes 7 and 8 (8505c-derived CM) and 9 and 10 (KTC-2-derived CM); quantitative analysis shown in Fig. 2F]. Altogether, these results suggest that ATC cells-derived CM induced an activated phenotype in MRC-5 cells similar to that induced by co-cultures and that ATC cells-derived CM can be used to gain additional insights on the effects of ATC cells-secreted factors on MRC-5 cell phenotype.

### ATC cells-derived CM stimulates cell proliferation of MRC-5 cells through the activation of Src and Akt pathways

**Figure 3:**
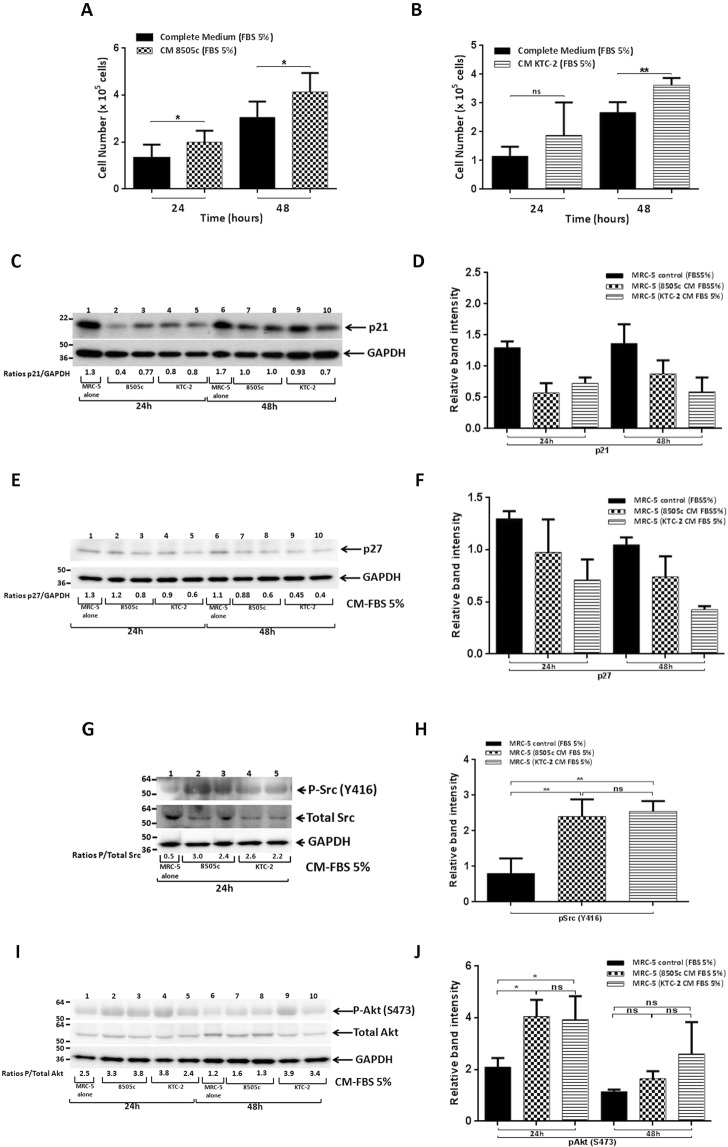
ATC cells-derived CM increased proliferation of MRC-5 cells. Proliferation of MRC-5 cells, incubated with 8505c-derived CM (**A**) or KTC-2-derived CM (**B**) for 24 h or 48 h, estimated by cell counting. Data are expressed as mean ± SD of 4 independent experiments (n = 4) with triplicate samples for each experimental group. Immunoblot analysis of p21 in MRC-5 cells grown under normal conditions (control) or exposed to CM derived from ATC cells, 8505c and KTC-2, for 24 h and 48 h (**C**). The figure shows a representative Western Blot of 3 independent experiments (n = 3) with duplicate samples for each experimental group. Quantification of relative expression of p21 protein in human fibroblasts after using GAPDH as loading control [(**D**) full blot is shown in the Supplemental Information, Full Original Blots-III]. The same blot was stripped and re-blotted using anti-GAPDH antibody for loading control. Immunoblot analysis of p27 in MRC-5 cells grown under normal conditions (control) or exposed to CM derived from ATC cells, 8505c and KTC-2, for 24 h and 48 h (**E**). The figure shows a representative Western Blot of 3 independent experiments (n = 3) with duplicate samples for each experimental group. Quantification of relative expression of p27 protein in human fibroblasts after using GAPDH as loading control [(**F**); full blot is shown in the Supplemental Information, Full Original Blots-III]. The same blot was cut and blotted using anti-GAPDH antibody for loading control. Immunoblot analysis of Src in MRC-5 cells grown under normal conditions (control) or exposed to CM derived from ATC cells, 8505c and KTC-2, for 24 h (**G**). The figure shows a representative Western Blot of 4 independent experiments (n = 4) with duplicate samples for each experimental group. Quantification of relative expression of pSrc/Total Src proteins in human fibroblasts [(**H**); full blot is shown in the Supplemental Information, Full Original Blots-III]. The same blot was stripped and re-blotted using anti-Total Src antibody. Data are expressed as mean ± SD. Immunoblot analysis of Akt in MRC-5 cells grown under normal conditions (control) or exposed to CM derived from ATC cells, 8505c and KTC-2, for 24 h and 48 h (**I**). The figure shows a representative Western Blot of 3 independent experiments (n = 3) with duplicate samples for each experimental group. Quantification of relative expression of pAkt/Total Akt proteins in human fibroblasts [(**J**); full blot is shown in the Supplemental Information, Full Original Blots-III]. The same blot was stripped and re-blotted using anti-Total Akt antibody. Data are expressed as mean ± SD. *p < 0.05 and **p < 0.005. ns, not significant.

Previous reports indicate that, in addition to the increased protein levels of markers of the CAF-phenotype, once activated, fibroblasts also increase their rate of proliferation^7,8,13^. We therefore examined cell proliferation of MRC-5 cells, first by counting cells after treatment with ATC cells-derived CM. As shown in Fig. 3A, MRC-5 cell number was significantly increased by treatment with 8505c-derived CM at 24 h and 48 h. Similar results were obtained by stimulation with KTC-2-derived CM (Fig. 3B).

We also examined the effects of ATC cells-derived CM on MRC-5 cells proliferation by 3-(4,5-dimethylthiazol-2-yl)-2,5-diphenyl-tetrazolium bromide (MTT) assay. Consistent with the increases in the cell number, MRC-5 cell proliferation was significantly increased after treatment with 8505c- and KTC-2-derived CM at 24 h and 48 h (Fig. S1, a and b).

The findings that ATC cells-derived CM modified cell proliferation of MRC-5 cells prompted us to evaluate the abundance of key regulators of cell cycle. In agreement with the stimulation of proliferation, Western Blot assays showed that the protein levels of cyclin-dependent kinase inhibitor, p21, was lower by ~1.5-fold in MRC-5 cells incubated for 48 h with 8505c-derived CM [Fig. 3C, compare lane 6 (control with 5% FBS), with lanes 7 and 8]. We also found a decrease in the protein levels of p21 by ~2.5-fold in MRC-5 cells when KTC-2 CM were used [Fig. 3C, compare lane 6 (control with 5% FBS), with lanes 9 and 10]. The band intensities were quantified and the extents of changes are shown in Fig. 3D.

We next determined another essential cell cycle regulator, p27. Figure 3E, shows that the p27 protein levels were also lower by ~1.4-fold in MRC-5 cells treated with 8505c-derived CM at both times analyzed [Fig. 3E, compare lanes 1 (24 h) and 6 (48 h) (controls with 5% FBS) with lanes 2 and 3 (24 h) and 7 and 8 (48 h)]. Consistently, the abundance of p27 was decreased by ~1.8- and 2.4-fold in MRC-5 cells incubated with KTC-2-derived CM at 24 h and 48 h respectively [Fig. 3E, compare lanes 1 (24 h) and 6 (48 h) (controls with 5% FBS) with lanes 4 and 5 (24 h) and 9 and 10 (48 h)]. The intensities of the bands in Fig. 3E were determined, and the graph is shown in Fig. 3F. These results indicate that the effect/s of secreted factors from ATC cells on fibroblasts cell proliferation is mediated, at least in part, by modulation of the regulators of the G1-S cell cycle.

The Src family of protein tyrosine kinases is a key regulator of broad cellular functions, including cell proliferation and cell growth through the activation of multiple signaling pathways. In addition, it has been reported the involvement of Src in the activation of fibroblasts^25^. To determine whether the decreased abundance of p21/p27 together with the observed phenotypic changes in human fibroblasts were also accompanied by the over-activation of Src in MRC-5 cells incubated with ATC cells-derived CM, we analyzed the protein levels of phosphorylated Src (p-Src) and total Src (Fig. 3G). Consistently, we found a marked upregulation of Src phosphorylated in MRC-5 cells incubated for 24 h with 8505c- and KTC-2-derived CM as compared with MRC-5 cells treated with DMEM-FBS 5% (controls), without major modifications in total Src [Fig. 3G, compare lane 1 (control) with lanes 2 and 3 (8505c-derived CM) and 4 to 5 (KTC-2-derived CM)]. The quantitative data of the band intensities are shown in Fig. 3H. These observations suggest that Src pathway is affected in fibroblasts after exposure to ATC cells-derived CM and that it could play a crucial role in cell proliferation as well as in the activation of MRC-5 cells.

It has been reported that Src regulates cell processes via activation of multiple downstream signaling pathways including phosphoinositide 3-kinase (PI3K)/protein kinase B (Akt). Besides, many studies have shown that Akt is involved in the regulation of proliferation through alteration in changing of the expression of key regulators^26^. To determine whether the decreased abundance of p21/p27 was also accompanied by the over-activation of Akt in MRC-5 cells incubated with ATC cells-derived CM, we determined the protein levels of phosphorylated (p-Akt) and total Akt (Fig. 3I). The ratios of p-Akt to total Akt levels indicate that there was an ~1.8- and 1.4-fold increase in the ratios of p-Akt to total Akt in MRC-5 cells treated with 8505c-derived CM in a time-dependent manner [Fig. 3I, compare lanes 1 (24 h) and 6 (48 h) (controls with 5% FBS), with lanes 2 and 3 (24 h) and 7 and 8 (48 h)]. We obtained similar results for MRC-5 by using CM obtained from KTC-2 cells [Fig. 3I, compare lanes 1 (24 h) and 6 (48 h) (controls with 5% FBS) with lanes 4 and 5 (24 h) and 9 and 10 (48 h)]. The quantitative data of the band intensities are shown in Fig. 3J, indicating that in addition to accelerating the G1-S cell cycle progression, the activated Akt signaling induced by ATC cells-derived CM treatment to fibroblasts also contributes to higher cell proliferation rate in human fibroblasts.

Mitogen-activated protein kinases (MAPKs) are a widely conserved family of serine/threonine protein kinases involved in many cellular programs, such as cell proliferation among others. Thus, we evaluated whether ATC cell-derived CM stimulated the activity of ERK1/2 and their possible contribution to cell proliferation observed in MRC-5 cells. We did not detect any changes in phosphorylated ERK1/2 under the same experimental conditions (Fig. S2).

Altogether, these results provide additional evidence that paracrine signaling within ATC cells-derived CM promoted MRC-5 proliferation through the activation of the Src kinase via, at least in part, Akt signaling and does not alter ERK 1/2 signaling.

### MRC-5 cells activated by ATC cells-derived CM enhanced their synthetic and secretory functions

**Figure 4:**
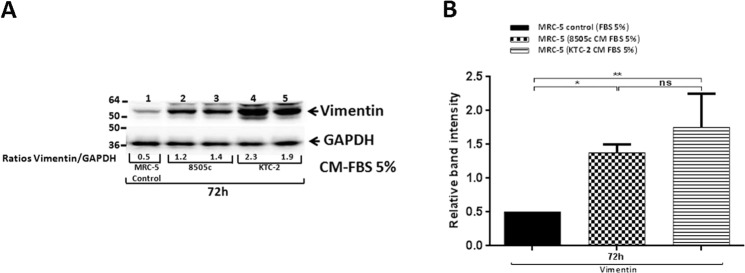
MRC-5 cells activated by ATC cells-derived CM enhanced their synthetic and secretory functions. Immunoblot analysis of vimentin in MRC-5 cells grown under normal conditions (control) or exposed to CM derived from ATC cells, 8505c and KTC-2, for 72 h (**A**). The figure shows a representative Western Blot of 3 independent experiments (n = 3) with duplicate samples for each experimental group. Quantification of relative expression of vimentin protein in human fibroblasts after using GAPDH as loading control [(**B**) full blot is shown in the Supplemental Information, Full Original Blots-IV]. The same blot was stripped and re-blotted using anti-GAPDH antibody for loading control. Data are expressed as mean ± SD. *p < 0.05 and **p < 0.01.

Reprogramming fibroblasts into CAFs also involves changes in synthetic and secretory functions of those cells^7,8,13^. Within those paracrine alterations, activated fibroblasts are known to gain the expression of, among other proteins, vimentin. Consistently, Western Blot analysis revealed an up-regulation of the mesenchymal marker vimentin in MRC-5 cells after treatment for 72 h with 8505c-derived CM [Fig. 4A, compare lanes 1 (controls with 5% FBS), with lanes 2 and 3]. We obtained similar results for MRC-5 by using CM obtained from KTC-2 cells [Fig. 4A, compare lanes 1 (controls with 5% FBS) with lanes 4 and 5]. The quantitative data of the band intensities are shown in Fig. 4B.

Given the robust role of IL-6 as a pro-tumorigenic cytokine in several types of cancer, we therefore assayed alterations in their levels. In keeping with our previous observations (Fig. 1F–I), we observed that the treatment of MRC-5 cells for 48 h with CM derived from 8505c cells as well as KTC-2 cells induced a strong release in the secretion of IL-6 at 24 and 48 h (Fig. S3). Taken together, our data indicate that paracrine factors released from human ATC cells reprogram the secretory phenotype of human fibroblasts and activate them into CAFs.

### The inhibition of ROS altered the metabolic reprogramming of MRC-5 cells triggered by ATC cells-derived CM, but no effect on the expression of IL-6 mRNA

**Figure 5:**
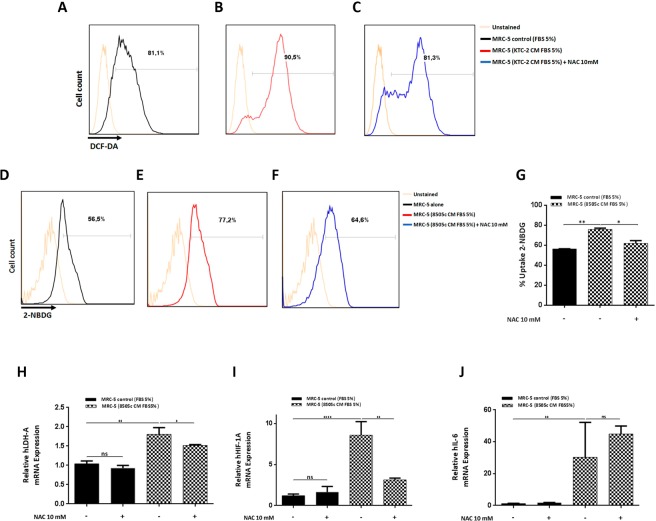
ATC cells-derived CM increased glucose uptake through ROS generation, in MRC-5 cells. Total intracellular levels of ROS in MRC-5 by flow cytometry. Representative histograms are shown of 2 independent experiments (n = 2) with triplicate samples for each experimental group. (**A**–**C**) Basal ROS production in MRC-5 cells grown under normal conditions (**A**) and incubated with KTC-2-derived CM for 24 h in the absence (**B**) or presence of the ROS scavenger NAC (10 mM) (**C**). Glucose uptake using the fluorescent glucose analog, 2-NBDG, followed by flow cytometry. Representative histograms (**D**–**F**) of 2 independent experiments (n = 2) with triplicate samples for each experimental group, and quantification (**G**) are shown. Data are expressed as mean ± SD. Basal glucose uptake in MRC-5 cells grown under normal conditions (**D**) and incubated with 8505c-derived CM for 24 h in the absence (**E**) or presence of the ROS scavenger NAC (10 mM) (**F**). Expression levels of LDH-A (**H**), HIF-1A (**I**) and IL-6 (**J**). mRNA levels by RT-qPCR in MRC-5 grown under normal conditions and incubated with 8505c-derived CM for 24 h in the absence or presence of NAC (10 mM). Data are expressed as mean ± SD of 3 independent experiments (n = 3) with triplicate samples for each experimental group. *p < 0.05, **p < 0.005 and ****p < 0.0001. ns, not significant.

Our data indicated that during co-culturing in transwell chambers with 8505c cells, the intracellular ROS levels in MRC-5 cells were significantly increased (Fig. 1D and E). We investigated the involvement of ROS on the activated-phenotype of human normal fibroblasts. We first analyzed the effect of ATC cells-derived CM on the intracellular ROS levels in MRC-5 cells. Consistent with the transwell system, MRC-5 cells incubated for 24 h with CM derived from KTC-2 cells show an increased in ROS production compared with complete media (Fig. 5, compare A and B), whereas this increase in ROS following the combination treatment was blocked in the presence of the ROS scavenger, N-acetyl-L-cysteine (NAC, 10 mM) (Fig. 5C). Similar results were obtained by stimulation with 8505c-derived CM (data not shown).

To further investigate ROS effects on the metabolic reprogramming of human normal fibroblasts, we next evaluated the glucose uptake in MRC-5 cells in response to ATC cells-derived CM. Consistent with our previous findings of increased expression of GLUT-1 in MRC-5 cells during co-culturing with ATC cells (Fig. 1L and 1M), glucose incorporation significantly increased in MRC-5 cells incubated for 24 h with 8505c-derived CM compared with complete media [Fig. 5, compare D, E and G (quantitative analysis)]. We further evaluate this effect in the presence of NAC and observed a significant decrease in glucose uptake after combination treatment of ROS scavenger with 8505c-derived CM [Fig. 5, compare E, F and G (quantitative analysis)].

We next examined the transcription of the glycolytic enzyme, LDH-A, following NAC treatment. Similar to the transwell system, treatment with CM derivate from 8505c cells, significantly increased their mRNA levels in MRC-5 cells at 24 h (Fig. 5H). Most interestingly, the combination treatment with 10 mM NAC, inhibited the increase in the mRNA levels of LDH-A (Fig. 5H). Similar results were obtained by stimulation with KTC-2-derived CM (data not shown).

As was previously mentioned, the enhanced glucose uptake and glycolysis is partly due to highly expressed HIF-1α. Therefore, we next explored the effect of NAC on the mRNA levels of HIF-1α. As determined by qPCR, we found that combination treatment of 8505c-derived CM and 10 mM NAC, significantly reduced the increase in the mRNA levels of HIF-1α in MRC-5 cells (Fig. 5I). In contrast, the combination treatment of 8505c-derived CM and 10 mM NAC did not alter the mRNA levels of IL-6 in MRC-5 cells (Fig. 5J). These data indicate that the ROS was increased in human fibroblasts by ATC cells-derived CM, positively modulates glucose metabolism through regulation of HIF-1α. On the other hand, the increased expression of IL-6 mRNA levels induced by 8505c-derived CM was independent of ROS.

### Induction of CAF-marker protein expression in human thyroid fibroblasts during co-cultures

**Figure 6:**
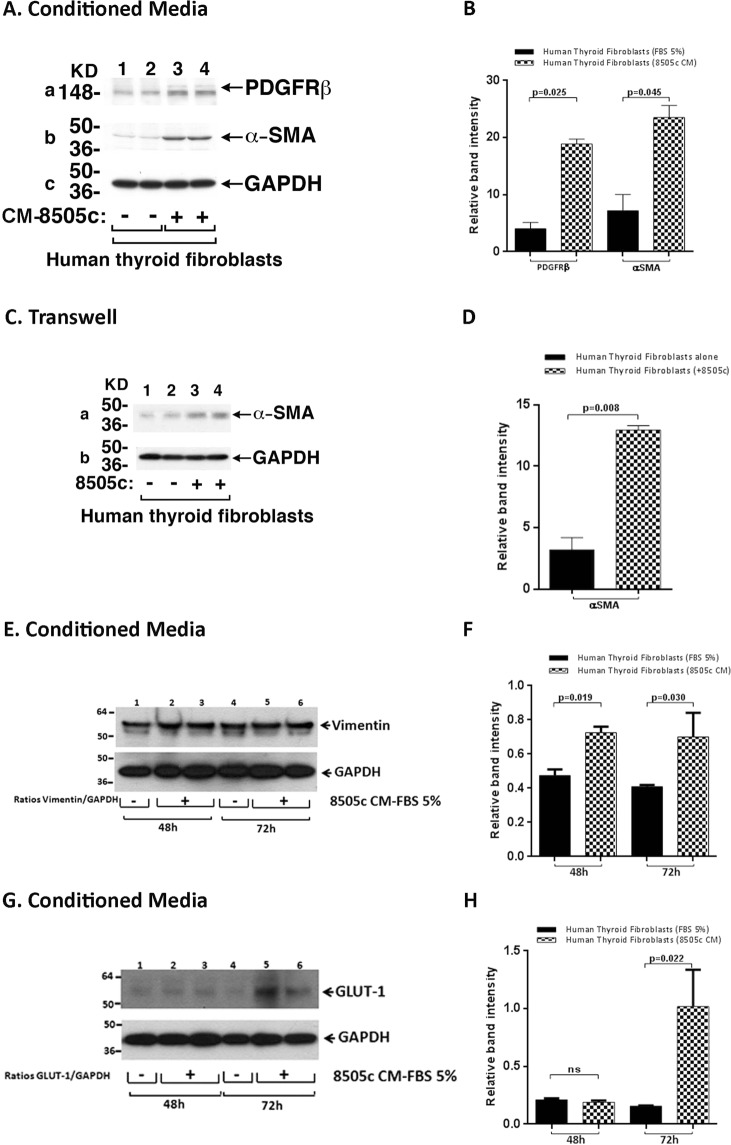
Co-cultures of ATC cells with fibroblasts induce the protein expression of CAF-markers in human thyroid fibroblasts. Effects of CM from 8505c cells on CAFs markers in human thyroid fibroblasts (**A**–**D**). (**A**) Immunoblot analysis of PDGFR-β (a) and α-SMA (b) in human thyroid fibroblasts, grown under normal conditions (lanes 1 and 2) or exposed to CM derived from 8505c cells for 48 h (lanes 3 and 4). The figure shows a representative Western Blot of 2 independent experiments (n = 2) with duplicate samples for each experimental group. (**B**) Quantification of relative expression of PDGFR-β and α-SMA proteins in human thyroid fibroblasts after using GAPDH as loading control. [Full blots are shown in the Supplemental Information, Full Original Blots-VI]. Data are expressed as mean ± SD. (**C**) Immunoblot analysis in human thyroid fibroblast after 48 h of co-cultures with ATC cells. Immunoblot analysis of α-SMA (a) in mono-cultures of human thyroid fibroblast (lanes 1 and 2) or after co-cultures with 8505c cells (lanes 3 and 4) in 12-well transwell system, for 48 h. The figure shows a representative Western Blot of 2 independent experiments (n = 2) with duplicate samples for each experimental group. (**D**) Quantification of relative expression of α-SMA protein in human thyroid fibroblasts after using GAPDH as loading control (Full blots are shown in the Supplemental Information, Full Original Blots-VI). Data are expressed as mean ± SD. (**E**) Immunoblot analysis of vimentin in human thyroid fibroblasts grown under normal conditions (lanes 1 and 4) or exposed to CM derived from 8505c for 48 h (lanes 2 and 3) and 72 h (lanes 5 and 6). The figure shows a representative Western Blot of 2 independent experiments (n = 2) with duplicate samples for each experimental group. (**F**) Quantification of relative expression of vimentin protein in human thyroid fibroblasts after using GAPDH as loading control. The same blot was stripped and re-blotted using anti-GAPDH antibody for loading control (Full blots are shown in the Supplemental Information, Full Original Blots-VI). Data are expressed as mean ± SD. (**G**) Immunoblot analysis of GLUT-1 in human thyroid fibroblasts grown under normal conditions (lanes 1 and 4) or exposed to CM derived from 8505c for 48 h (lanes 2 and 3) and 72 h (lanes 5 and 6). The figure shows a representative Western Blot of 2 independent experiments (n = 2) with duplicate samples for each experimental group. (**H**) Quantification of relative expression of GLUT-1 protein in human thyroid fibroblasts after using GAPDH as loading control. The same blot was stripped and re-blotted using anti-GAPDH antibody for loading control. [Full blots are shown in the Supplemental Information, Full Original Blots-VI]. Data are expressed as mean ± SD.

We next tested whether human thyroid fibroblasts would be similarly activated as MRC-5 cells did by ATC cells. Figure 6A shows the activation markers, PDGFR-β (panel a) and α-SMA (panel b) were elevated in human thyroid fibroblasts when cultured in the 8505c-derived CM [Fig. 6A, a and b: compare lanes 1 and 2 (controls) with lanes 3 and 4, respectively; also see the quantitation graph, Fig. 6B]. α-SMA was also shown to be elevated in human thyroid fibroblasts by the transwell assays [Fig. 6C, compare lanes 1 and 2 (controls) with lanes 3 and 4; see also quantitative data shown in Fig. 6D].

We next further analyzed the CM derived from ATC cells could also activate the expression of vimentin, an EMT marker, in human thyroid fibroblasts as in MRC-5 cells. Similar to that shown for MRC-5 cells (see Fig. 4A and 4B), we found that vimentin was significantly elevated after treatment of human thyroid fibroblasts with 8505c-derived CM [Fig. 6E, compare lanes 1 and 4 (controls) with lanes 2 and 3 (48 h) and lanes 5 and 6 (72 h); see also quantitative data shown in Fig. 6F]. The glucose transporter 1, GLUT-1, was also markedly increased in human thyroid fibroblasts cultured in the CM derived from 8505c cells at 72 h, as shown for MRC-5 cells [Fig. 6G, compare lane 4 (control) with lanes 5 and 6 (72 h); see also quantitative data shown in Fig. 6H]. The activation of similar markers (PDGFR-β, α-SMA, vimentin and GLUT-1) suggested that ATC cells could activate human thyroid fibroblasts as in MRC-5 cells.

### Induction of epithelial-to-mesenchymal transition (EMT) by treatment with CM from activated fibroblasts to increase proliferation and invasion of follicular FTC-133 cells

**Figure 7:**
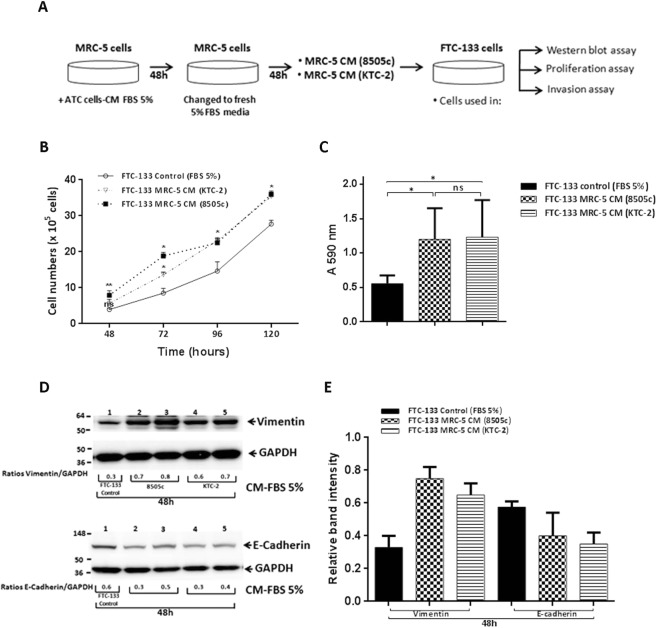
Treatment with CM from activated fibroblasts increased proliferation and invasion of FTC-133 cells. (**A**) Schematic representation of CM preparation from MRC-5 cells and treatment of FTC-133 cells. Cell proliferation (**B**) and *in vitro* invasion assays (**C**) were performed in FTC-133 cells incubated with CM derived from activated MRC-5 cells, as described in the Materials and Methods section. Data are expressed as mean ± SD of 3 independent experiments (n = 3) with quadruplicate samples for each experimental group. (**D**) Immunoblot analysis of vimentin, E-cadherin and GAPDH as a loading control after treatment with CM derived from activated MRC-5 cells. The figure shows representative Western Blots of 3 independent experiments (n = 3) with duplicate samples for each experimental group. (**E**) Quantification of relative expression of proteins associated with EMT in FTC-133 after using GAPDH as loading control (Full blots are shown in the Supplemental Information, Full Original Blots-VII). Data are expressed as mean ± SD.*p < 0.05 and **p < 0.01.

Activated fibroblasts have been reported to affect tumor malignancy mainly through secretion of cytokines, chemokines, and growth factors, thereby enhancing cancer cell proliferation and invasive properties. We therefore next explored the functional effects of soluble factors secreted from MRC-5 cells after activation with ATC cells-derived CM in thyroid carcinogenesis. To do so, we collected CM from MRC-5 cells cultured with CM of 8505c and KTC-2 cells for 48 h. After 48 h, we replaced media to fresh media containing 5% FBS for an additional 48 h. The collected supernatant [(labeled as MRC-5 CM (ATC)] was then used to treat FTC-133 cancer cells for analyses of cell proliferation (Fig. 7A). The results showed that thyroid cancer cells treated with the CM from MRC-5 activated with 8505c-derived CM increased the proliferation in a time-dependent manner (Fig. 7B). Similar results in FTC-133 cell proliferation were obtained by using CM from MRC-5 activated with KTC-2-derived CM (Fig. 7B).

To investigate whether CM from MRC-5 activated by ATC-derived CM affected invasion of cells, we carried out cell invasion assays. Importantly, cell invasiveness of FTC-133 cells was significantly increased after treatment with CM of fibroblasts induced for 48 h with ATC cells-derived CM, compared to control (Fig. 7C).

We next focused on the analysis of regulators affecting cell motility and migration. We examined the markers of EMT after the treatment of FTC-133 cells with CM from MRC-5 activated by ATC-derived CM. As shown in Fig. 7D, treatment with MRC-5 CM led to an increase of vimentin protein abundance as compared with FTC-133 control cells (see also the quantification in Fig. 7E). We also determined the levels of other EMT marker, E-cadherin, because its repressed expression is a hallmark of EMT. Indeed, E-cadherin was decreased after treatment with CM from MRC-5 activated by ATC-derived CM compared with controls FTC-133 cells (Fig. 7D, see also the quantification in Fig. 7E). Taken together, these data indicate that CM from MRC-5 activated by ATC cells-derived CM increased tumor cell proliferation, invasion and activation of EMT.

## Discussion

**Figure 8:**
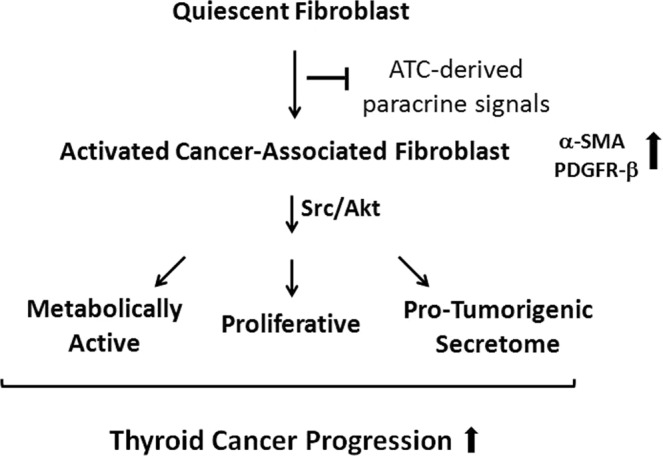
Proposed model describing the paracrine loop existing between anaplastic thyroid tumor cells and stromal fibroblasts in TC. Soluble signals derived from ATC cells promotes the up-regulation of pSrc and pAkt in human fibroblasts, leading to increased cell proliferation, secretory functions and metabolic reprogramming of fibroblasts, consistent with the induction of CAF phenotype. Moreover, CM from activated fibroblasts promoted thyroid cell proliferation and invasion, thereby increasing TC progression.

The role of neoplastic epithelial cells in tumorigenesis, including thyroid cancer, has been extensively investigated for several decades. However, less attention has been paid to the study of the surrounding TME in these events. It has been demonstrated that the TME, which is composed of many stromal cells (particularly fibroblasts), plays a key role in tumor progression^7,8,14^. Consequently, in the present study, we analyzed the interplay between fibroblasts and ATC cells and how these interactions can promote TC. We demonstrated the function of tumor-secreted factors from two well-characterized ATC cell lines on the reprogrammed-phenotype of human normal fibroblasts, and found that the treated quiescent fibroblasts with ATC cells-derived CM became more proliferative, acquired an active secretome and were more active metabolically, with all these changes being related to a phenotype of activated-fibroblast or CAF^7,13^. The reprogramming of quiescent fibroblasts into CAFs was further confirmed by the up-regulation of PDGFR-β and α-SMA, both acknowledged markers of CAFs. These effects were mediated, at least in part, through the activation of Src and its downstream target Akt, in fibroblasts. Finally, the secretome from activated-fibroblasts led to significant changes in the proliferation and invasion of follicular thyroid cancer cells (Fig. 8). Taken together, our findings implicate secreted factors from activated fibroblasts by ATC cells-derived CM, as being a key event in TC progression.

Previous studies have shown that tumor cells secrete multiple factors, including growth factors and cytokines, among others, which drive the reprogramming of fibroblasts into CAFs^8,12^. By focusing on the anaplastic thyroid tumor cell secretome effects exerted in the immediate proximity of stromal cells, we demonstrated that soluble factors secreted by ATC cells induced fibroblast activation in both transwell co-cultures as well as with ATC cells-derived CM. In fact, the release of ROS by tumor cells is known to promote the activation of fibroblasts in many tumor types, and several studies have highlighted the role of ROS in the conversion of quiescent fibroblasts into activated-fibroblasts, through an increase of CAF markers. In contrast, antioxidants have been found to inhibit these activated-fibroblast features^8,16^. In agreement, we found that ROS are abundantly produced by mono-cultures of 8505c cells, suggesting that ROS could be one of the tumor signals involved in the phenotypic reprogramming of fibroblasts, with a co-culture with 8505c cells as well as incubation with ATC cells-derived CM inducing the generation of high levels of ROS in MRC-5 cells (Figs 1D and E and 5B). Here we also demonstrated antioxidant NAC attenuate glucose uptake in human fibroblasts, after having been treated with ATC cells-derived CM (Fig. 5F and G). Importantly, this effect is partially mediated by inhibition of HIF-1α (Fig. 5I), and a reduction in the HIF-1α mRNA expression level may lead to a decrease in the transcription of the glucose metabolism-related genes LDH-A (Fig. 5H) and GLUT-1. Consequently, given that HIF-1α undergoes posttranscriptional regulation^27^, HIF-1α protein modifications would be expected to occur. These results suggest that increases in ROS production is partially involved in the metabolic reprogramming mediated by ATC cells-derived CM and support our proposal that ROS could be one of the positive mediators involved in fibroblast activation.

In addition to ROS, cytokines have been shown to be released by cancer cells and affect the differentiation of fibroblasts. In particular, IL-6 is also known to elicit a fibroblast activated phenotype, and consistent with these results, increased IL-6 basal levels secreted by KTC-2 cells were found. Thus, in our study, fibroblast activation and differentiation may be in part mediated by the inflammatory cytokine IL-6, secreted from ATC cells, as well as by ROS. However, further studies are needed to investigate their participation. Moreover, we cannot rule out other paracrine mediators derived from ATC cells being responsible for the phenotypic and functional changes of human fibroblasts. Taken together, our results suggest a fibroblast differentiation to CAFs induced by ATC cells-derived CM.

Reprogramming fibroblasts into CAFs involves the activation of multiple signaling pathways, with previous studies having implicated the Src family kinase pathway as being essential for myofibroblast differentiation^25^. Our findings suggest that the Src pathway is involved in MRC-5 cells proliferation, differentiation and activation after ATC cells-derived CM treatment. The Src tyrosine kinases are redox regulated proteins and we have observed that large amounts of oxidants are produced by anaplastic thyroid tumor cells. We speculate that the activation of Src in MRC-5 cells during co-cultures may be mediated by ATC cells-derived ROS, as has been previously described by others, resulting later in the activation of the Akt pathway. Based on these data, we hypothesize that Src kinase regulates fibroblast activation, at least in part, through an Akt-dependent pathway. Further studies are needed to explore other ATC cell-derived factors, as well as the pathways involved in phenotypic reprogramming of human fibroblasts.

Once transformed into CAFs, activated fibroblasts reprogram their metabolic and their secretory phenotype to enhance tumor progression^8,17^, thus provoking the question of whether these metabolic shifts, as well as factors secreted by activated fibroblasts with ATC cells-derived factors, could regulate TC proliferation and invasion. In this regard, we observed that treatment with CM of fibroblasts activated by ATC cells-derived CM promoted cell proliferation and invasion of the follicular thyroid cancer cell line, FTC-133 (Fig. 7), with it being clear that these effects were mediated by soluble factors produced by activated fibroblasts. Our results demonstrated that activated fibroblasts produce and secrete very high levels of IL-6, which could represent a key candidate factor for increasing FTC-133 cell proliferation and invasion. However, we speculate that IL-6 is not the only factor involved in these events.

The cellular components of the thyroid tumor stroma include, among others, endothelial cells and immune cells, in addition to activated fibroblasts. In the current study, we focused our analysis on activated fibroblast-derived signals on thyroid tumor progression. Nevertheless, we cannot rule out the possibility that secretome from other cell components of the TME could also play a major role in TC progression, thereby contributing to thyroid carcinogenesis. Further studies exploring how these components may work together to promote anaplastic thyroid carcinogenesis could help to address this issue in the future.

The recruitment of fibroblasts in mouse models of papillary thyroid cancer was previously reported^28,29^. However, there are still many unanswered questions, such as which oncogenic signals are responsible for the recruitment of fibroblasts into thyroid TME and how they act. Further *in vivo* studies would be helpful to elucidate this key event.

In conclusion, our study provides evidence that human ATC cells-derived soluble factors contribute to the phenotypic, secretory and metabolic reprogramming of human fibroblasts, which favor TC development and progression. Taken together, our findings contribute to a better understanding of the molecular basis of interactions between anaplastic thyroid tumor cells and stromal cells. Furthermore, these data may lead to the identification and targeting of thyroid tumor-supportive mechanisms. However, whether or not this phenomenon occurs *in vivo* still remains to be clarified.

## Materials and Methods

### Cell cultures

Human fibroblasts, MRC-5 cells, were obtained from ATCC (ATCC CCL-171) and used at passage number ranged from 23 to 29. Human thyroid fibroblasts were purchased from ScienCell Research Laboratories (#3730).

Human ATC cells, 8505c and KTC-2 (passage number ranged from 10 to 15), were authenticated by short tandem repeat (STR) profiling analysis by the Science Cordoba Agency (CEPROCOR, Córdoba, Argentina). Briefly, samples were amplified by PowerPlex Fusion kit (Promega) and after capillary electrophoresis (GA 3130-Applied Biosystems), the data were analyzed with Gene Mapper Software v3.2 (Applied Biosystems). All cells were cultured in Dulbecco’s modified Eagle’s medium (DMEM) (Gibco) supplemented with 10% fetal bovine serum (FBS) (Hyclone), 100 units/mL penicillin, and 100 μg/mL streptomycin (Gibco). Human follicular thyroid cancer cells, FTC-133, were cultured in DMEM/ Ham’s F12 (1:1) medium (Gibco) supplemented with 10% FBS (Hyclone), 100 units/mL penicillin, and 100 μg/mL streptomycin (Gibco), as previously described^30^. FTC-133 cells retained differentiated thyrocyte function and thyrocyte responsiveness to thyrotropin and local active growth factors.

### Co-cultures in transwell system

For non-contact co-cultures, human MRC-5 cells were seeded (3 × 10^5^ or 1 × 10^5^ cells) in the lower wells of a transwell system (6-well or 12-well types respectively) and ATC cells were cultured in the upper chambers (cell culture inserts polyester membrane with 0.4 μm pores to allow the diffusion of the soluble factors only; Corning) (Fig. 1A). After 24 h and 48 h of incubation, the culture medium and cells were harvested and intracellular levels of ROS, secretion and expression of interleukin-6 (IL-6), expression of the hypoxia inducible transcription factor-1α (HIF-1α), glucose transporter 1 (GLUT-1), lactate dehydrogenase A (LDH-A), platelet-derived growth factor receptor–β (PDGFR-β) and α-smooth muscle actin (α-SMA) were evaluated by flow cytometry, quantitative real time reverse transcription polymerase chain reaction (RT-qPCR), enzyme-linked immunosorbent assay (ELISA) and Western Blotting respectively. Mono-cultures of MRC-5, 8505c and KTC-2 cells were processed as controls.

### Determination of intracellular levels of ROS

Cells were incubated with 10 μM 2′, 7′-dichlorodihydrofluorescein diacetate, (H_2_DCFDA, Molecular Probes) in phosphate buffered saline (PBS) (Gibco). After 30 min at 37 °C, cells were washed and re-suspended in saline solution with 2% FBS. Detection of ROS levels was carried out through flow cytometric analyses using a FACS Canto II flow cytometer (BD Biosciences) with FlowJo software (version 7.6.2).

### Determination of IL-6 by ELISA

Culture supernatants were harvested, clarified by centrifugation, and frozen for the subsequent determination of IL-6 concentration by ELISA according to the manufacturer’s instructions (#430501, eBiolegend).

### Quantification of platelet-derived growth factor (PDGF) by ELISA

Cell culture supernatant was collected 48 h and centrifuged to remove particulates before being stored at −80 °C. Culture medium from normal thyroid served as a control. Concentrations of PDGF in CM from each cell lines were measured using ELISA kits (abcam, ab100624 for sub-type PDGF-BB) according to the manufacturer’s instructions. And the absorbance was read at 450 nm with SpectraMax 250 micro titer plate reader. For wavelength correction, subtract readings at 570 nm from the readings at 450 nm.

### RNA Isolation and quantitative real-time RT-PCR

Total RNA purification, cDNA synthesis, and quantitative PCR (qPCR) were performed as previously described^31,32^. The specific primer sequences were as follows: hGLUT-1 forward, 5′-TCACTGTGCTCCTGGTTCTG-3′ and reverse, 5′-CCTGTGCTCCTGAGAGATCC-3′; hHIF-1A forward, 5′-AGCCAGATCTCGGCGAAGT-3′ and reverse, 5′-CAGAGGCCTTATCAAGATGCG-3′; hIL-6 forward, 5′-TCAATGAGGAGACTTGCCTG-3′ and reverse, 5′-TCATCTGCACAGCTCTGGCT-3′; hLDH-A forward, 5′-CAGGTGGTTGAGAGGGTCTTT-3′ and reverse, 5′-CTTCAAACGGGCCTCTTCCT-3′. hGAPDH forward, 5′-TTG TTG CCA TCA ATG ACC CCT T-3′ and reverse, 5′-CAG TGG ACT CCA CGA CGT ACT CAG-3′.

### Western Blot analysis

Whole-cell lysates was prepared as previously described^30^. The protein samples (20–30 µg) were analyzed by Western blot as described previously^32^. Antibodies used according to the manufacturers’ manuals include GLUT-1 (#12939) from Cell Signaling and used at a 1:600 dilution. PDGFR-β (sc-374573) and α-SMA (sc-53015) antibodies were purchased from Santa Cruz Biotechnology and used at a 1:200 dilution. Anti-p21 (#2947), anti-phosphorylated Akt (Ser473, #9271), Akt (#9272), phosphorylated Src (tyr416,#2101), total Src (#2110), vimentin (#5741), anti pErk1/2 (#9102) and GAPDH (#2118), all from Cell Signaling and used at a 1:1000 dilution. The antibody to p27 (#ab137736) (1:2000 dilution) was purchased from Abcam. Band intensities were quantified by using NIH IMAGE software (ImageJ 1.50i; NIH).

### Conditioned media (CM) harvesting

CM preparation from tumor cell lines: ATC cells (8505c and KTC-2) were seeded (1 × 10^6^ cells) on 100 mm tissue culture plates in DMEM 10% FBS. After 48 h incubation, the media were replaced with media with 5% FBS. After additional 48 h, CM were collected and clarified by centrifugation (2000 rpm for 10 min) and labeled as 8505c CM FBS 5% or KTC-2 CM FBS 5%, respectively. CM produced was used for the treatment (24h–72h) of MRC-5 cells (Fig. 2A).

CM from MRC-5 cells: To explore the effects of secreted factors present in the CM of MRC-5 cells after induction with ATC cells-derived CM on thyroid carcinogenesis, we generated CM by culturing fibroblasts (1.5 × 10^6^ cells on 100 mm plates) with ATC cells-derived CM for 48 h as described above. The media was replaced to fresh media containing 5% FBS. After 48 h, the supernatant was harvested [labeled as MRC-5 CM (ATC)], centrifuged and used for proliferation, migration and Western Blot assays of thyroid FTC-133 cells (Fig. 7A).

### MTT assay

The assay was optimized for the MRC-5 cells. Briefly, at the end of the incubation time, MRC-5 cells were incubated for at 37 °C for 4 h with 0.5 mg/ml of MTT, dissolved in serum free medium. Washing with PBS was followed by the addition of dimethyl sulfoxide (DMSO), gentle shaking for 10 min so that complete dissolution was achieved. Aliquots of the resulting solutions were transferred in 96-well plates and absorbance was measured at 540 nm using a microplate reader (Bio-Rad).

### Glucose uptake assays

MRC-5 cells were incubated for 2 h in 100 μl glucose-free media 1% FBS. Then, culture medium was removed from each well and replaced with 100 μl of culture medium in the absence or presence of the fluorescent 2-(*N*-(7-Nitrobenz-2-oxa-1,3-diazol-4-yl)Amino)-2-Deoxyglucose (2-NBDG) (10 ug/ml) (Invitrogen). Cells were cultured for 2 h before measuring fluorescence by flow cytometry (BD Biosciences).

### *In vitro* cell proliferation assay

Cell proliferation assay was performed as previously described with minor modifications^30^. Briefly, 5 × 10^4^ FTC-133 cells were seeded in six-well plates in medium supplemented with 1% FBS. Next day (day 0) cells were cultured in MRC-5 CM (8505c) or MRC-5 CM (KTC-2), replacing the MRC-5 CM (ATC) every 48 h (Fig. 7A). Nonattached cells were removed by gently washing twice with PBS. After trypsin treatment and resuspension, the number of attached cells was counted every 24 h from day 2–5 by using an automated cell counter (ThermoFisher Scientific).

### Invasion assay

Invasion assay was performed as described previously^33^ in 8-μm−pore transwells (Corning) in quadruplicate. Transwell filters were layered with 200 μL of Matrigel (BD Biosciences) diluted 1:10 in PBS. After rinsing with PBS, cells were plated in the upper chambers (5 × 10^4^ cells/24 well plates) and the MRC-5 CM (8505c) or MRC-5 CM (KTC-2) (Fig. 7A) in the lower wells (500 μL). 48 h later, cells migrating to the bottom of the filter were evaluated after removal of material from the upper side of the filter, with 0.1% crystal violet staining and measurement of solubilized dye at 590 nm.

### Statistical analysis

Analysis of intergroup differences was conducted by one-way analysis of variance (ANOVA), followed by the Turkey’s test and for the analysis of differences between two groups statistical analyses were performed using the Student’s t-test, with the use of GraphPad Prism 6.01 (GraphPad Software). Values of p < 0.05 were considered to be statistically significant.

## Supplementary information

Supplementary Information
