# Author Correction: Interplay of fibroblasts with anaplastic tumor cells promotes follicular thyroid cancer progression

**DOI:** 10.1038/s41598-019-54273-0

**Published:** 2019-12-05

**Authors:** Laura Fozzatti, Vanina Alejandra Alamino, Sunmi Park, Lucila Giusiano, Ximena Volpini, Li Zhao, Cinthia Carolina Stempin, Ana Carolina Donadio, Sheue-yann Cheng, Claudia Gabriela Pellizas

**Affiliations:** 1 Centro de Investigaciones en Bioquímica Clínica e Inmunología - Consejo Nacional de Investigaciones Científicas y Técnicas. Departamento de Bioquímica Clínica, Facultad de Ciencias Químicas, Universidad Nacional de Córdoba, Córdoba, Argentina; 2 Laboratory of Molecular Biology, Center for Cancer Research, National Cancer Institute, National Institutes of Health, Bethesda, Maryland USA

Correction to: *Scientific Reports* 10.1038/s41598-019-44361-6, published online 29 May 2019

The Acknowledgements section in this Article is incomplete.

“We thank M. P. Abadie and M. P. Crespo for their excellent technical assistance. We also thank Woo Kyung Lee of National Cancer Institute for providing the media from normal thyroid cells for the analysis of platelet-derived growth factor. The present research was supported by research grants from the Intramural Research Program at the Center for Cancer Research, (CCR, NCI, NIH), Consejo Nacional de Investigaciones Científicas y Técnicas (CONICET, PIP 112-20150100810), Agencia Nacional de Promoción Científica y Técnica (y PICT 2015-0340), Secretaría de Ciencia y Técnica (SeCyT, Universidad Nacional de Córdoba) and L.F. is grateful to CONICET to fund international activities.”

should read:

“We thank M. P. Abadie and M. P. Crespo for their excellent technical assistance. We also thank Woo Kyung Lee of National Cancer Institute for providing the media from normal thyroid cells for the analysis of platelet-derived growth factor. The present research was supported by research grants from the Intramural Research Program at the Center for Cancer Research, (CCR, NCI, NIH), Consejo Nacional de Investigaciones Científicas y Técnicas (CONICET, PIP 112-20150100810), Agencia Nacional de Promoción Científica y Técnica (PICT 2015-0340), Secretaría de Ciencia y Técnica (SeCyT, Universidad Nacional de Córdoba) and Fundación Sales. Also, L.F. is grateful to CONICET to fund international activities”

