# Supplementary material for: Interplay of fibroblasts with anaplastic tumor cells promotes follicular thyroid cancer progression
Source: Sci Rep. 2019 May 29;9:8028. doi: 10.1038/s41598-019-44361-6 (PMC6541589; doi:10.1038/s41598-019-44361-6)

Supplementary Information

Interplay of fibroblasts with anaplastic tumor cells promotes follicular thyroid cancer progression

Laura Fozzatti[1,2*], Vanina Alamino[1], Sunmi Park[2], Lucila Giusiano[1], Ximena Volpini[1], Li Zhao[2], Cinthia Stempin[1], Ana Donadio[1], Sheue-yann Cheng[2,3] and Claudia Gabriela Pellizas[1,3]

[1]Centro de Investigaciones en Bioquímica Clínica e Inmunología - Consejo Nacional de Investigaciones Científicas y Técnicas. Departamento de Bioquímica Clínica, Facultad de Ciencias Químicas, Universidad Nacional de Córdoba, Córdoba, ARGENTINA.

[2]Laboratory of Molecular Biology, Center for Cancer Research, National Cancer Institute, National Institutes of Health, Bethesda, Maryland, USA.

[*]Correspondence should be addressed to: Dr. Laura Fozzatti (lfozzatti@fcq.unc.edu.ar). Centro de Investigaciones en Bioquímica Clínica e Inmunología - Consejo Nacional de Investigaciones Científicas y Técnicas. Departamento de Bioquímica Clínica, Facultad de Ciencias Químicas, Universidad Nacional de Córdoba. Haya de la Torre y Medina Allende, X5000HUA Córdoba, ARGENTINA. Phone: +54 0351 5353851.

Supplementary Figure Legends

Figure S1. ATC cells-derived CM increased proliferation of MRC-5 cells.

Proliferation of MRC-5 cells, incubated with 8505c-derived CM (a) or KTC-2-derived CM (b) for 24h or 48h, estimated by MTT assay. Data are expressed as mean ± SD of 3 independent experiments (n=3) with quadruplicate samples for each experimental group. *p<0.05, **p< 0.005 and ****p< 0.0001.

Figure S2. ATC cells-derived CM did not modify mitogen-activated protein kinase (ERK1/2) pathway in human fibroblasts.

Immunoblot analysis of phosphorylated ERK1/2 and GAPDH as a loading control in MRC-5 cells grown under normal conditions (control) or exposed to CM derived from 8505c cells, for 24h and 48h. The figure shows a representative Western Blot of 3 independent experiments (n=3) with duplicate samples for each experimental group.

Figure S3. MRC-5 cells activated by ATC cells-derived CM enhanced their synthetic and secretory functions: Secreted IL-6 by ELISA.

MRC-5 cells were incubated with DMEM-FBS 5% medium (control), 8505c-derived CM or KTC-2-derived CM for 48h. The media was replaced to fresh media with 5% FBS. After 24h and 48h, the supernatant was harvested and used for ELISA assays. Data are expressed as mean ± SD of 3 independent experiments (n=3) with triplicate samples for each experimental group. ****p<0.0001.

Figure S1.

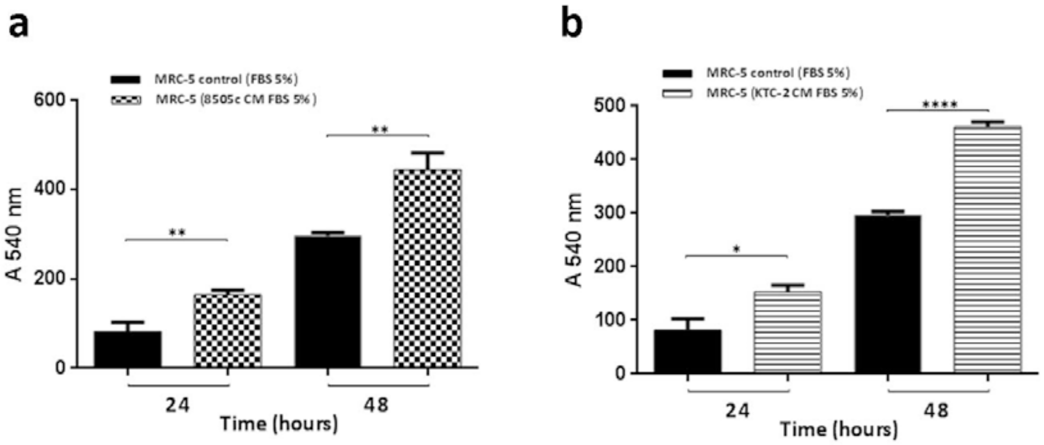
a
MRC-5 control (FBS 5%)
MRC-5 (8505c CM FBS 5%)
A 540 nm
600
400
200
0
**
**
24
48
Time (hours)
b
MRC-5 control (FBS 5%)
MRC-5 (KTC-2 CM FBS 5%)
A 540 nm
500
400
300
200
100
0
*
****
24
48
Time (hours)

Figure S2.

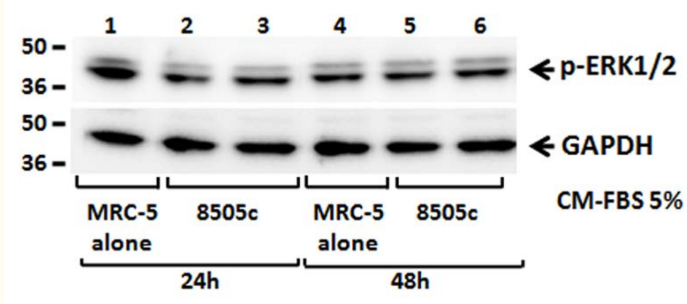
1
2
3
4
5
6
50
36
50
36
p-ERK1/2
GAPDH
MRC-5 alone
8505c
MRC-5 alone
8505c
CM-FBS 5%
24h
48h

Figure S3.

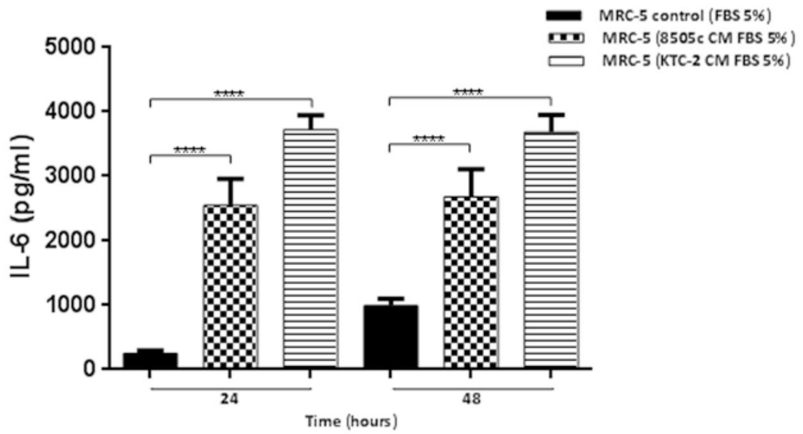
MRC-5 control (FBS 5%)
MRC-5 (8505c CM FBS 5%)
MRC-5 (KTC-2 CM FBS 5%)
5000
4000
3000
2000
1000
0
IL-6 (pg/ml)
****
****
****
****
24
48
Time (hours)

Full Original Blots-I .

Panel A: This figure displays the full original blots for Figure 1M shown in the text/Results. The identification of GLUT-1 bands was based on the expected molecular weight. The same blot was stripped and re-blotted using anti-GAPDH antibody for loading control.

Panel B: This figure displays the full original blots for Figure 1P and Q shown in the text/Results. The identification of PDGFR-β and α-SMA bands was based on the expected molecular weight. The same blot was stripped and re-blotted using anti-GAPDH antibody for loading control.

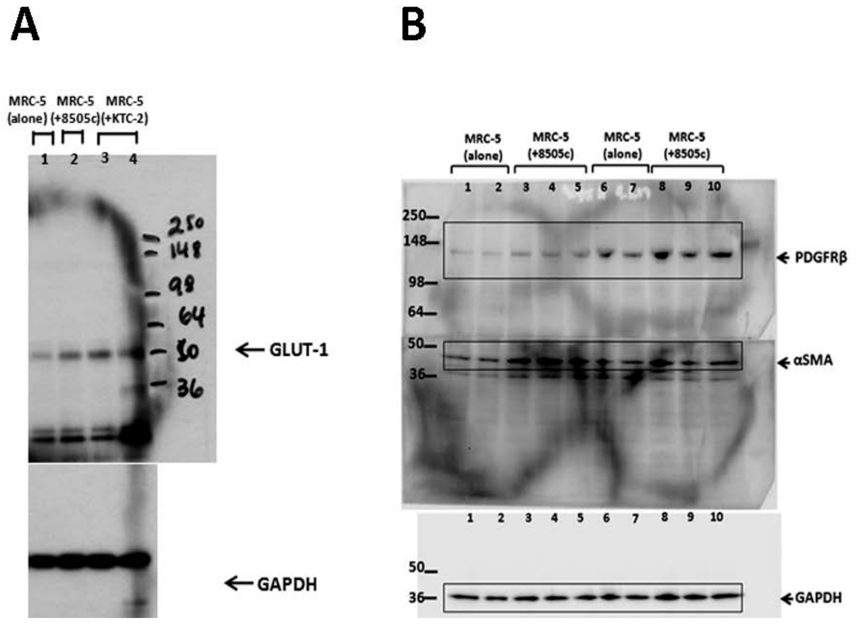

Full Original Blots-II.

Panel A: This figure displays the full original blots for Figure 2B shown in the text/Results. The identification of PDGFR-β bands was based on the expected molecular weight. The same blot was cut and blotted using anti-GAPDH antibody for loading control.

A

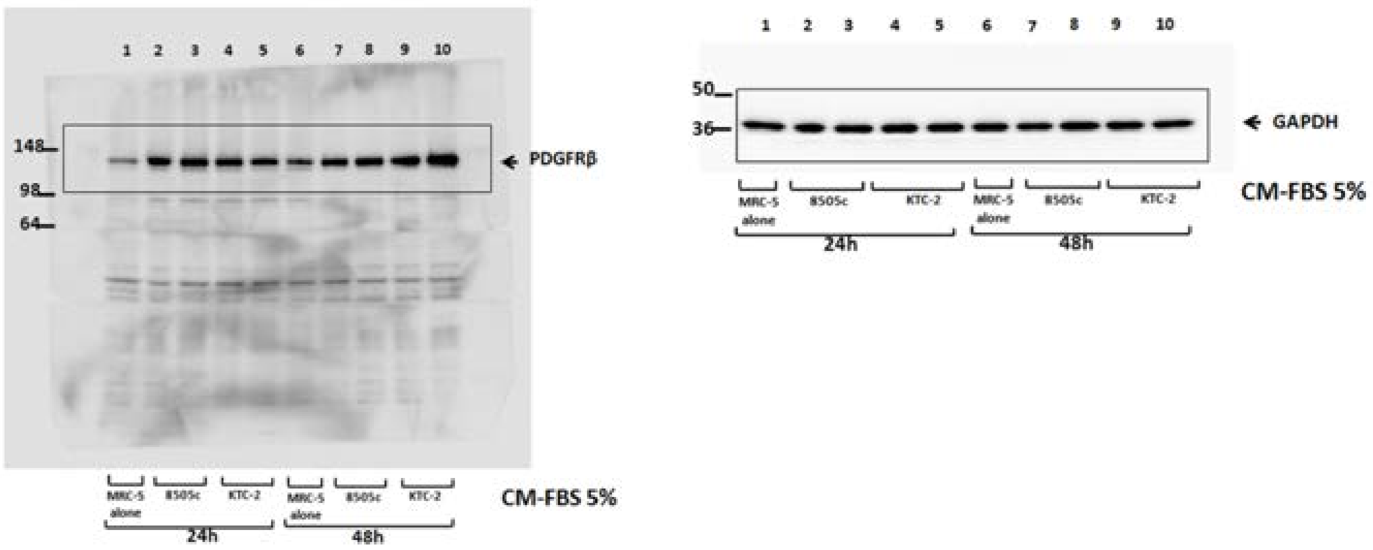

Full Original Blots-II.

Panel B: This figure displays the full original blots for Figure 2E shown in the text/Results. The identification of α-SMA bands was based on the expected molecular weight. The same blot was stripped and re-blotted using anti-GAPDH antibody for loading control.

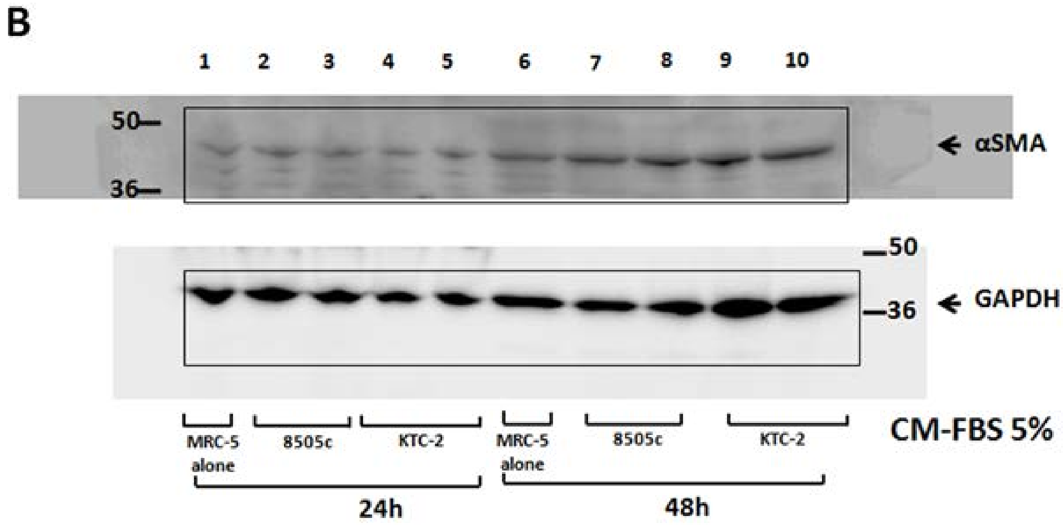

Full Original Blots-III.

Panel A: This figure displays the full original blots for Figure 3C shown in the text/Results. The identification of p21 bands was based on the expected molecular weight. The same blot was stripped and re-blotted using anti-GAPDH antibody for loading control.

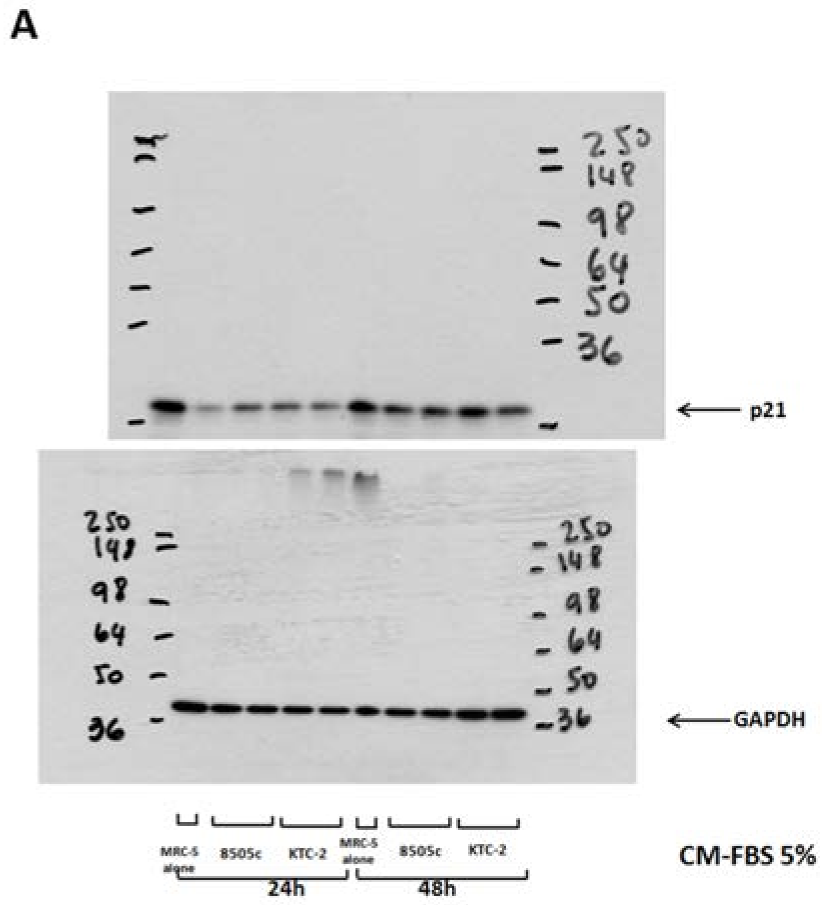

Full Original Blots-III.

Panel B: This figure displays the full original blots for Figure 3E shown in the text/Results. The identification of p27 bands was based on the expected molecular weight. The same blot was cut and blotted using anti-GAPDH antibody for loading control.

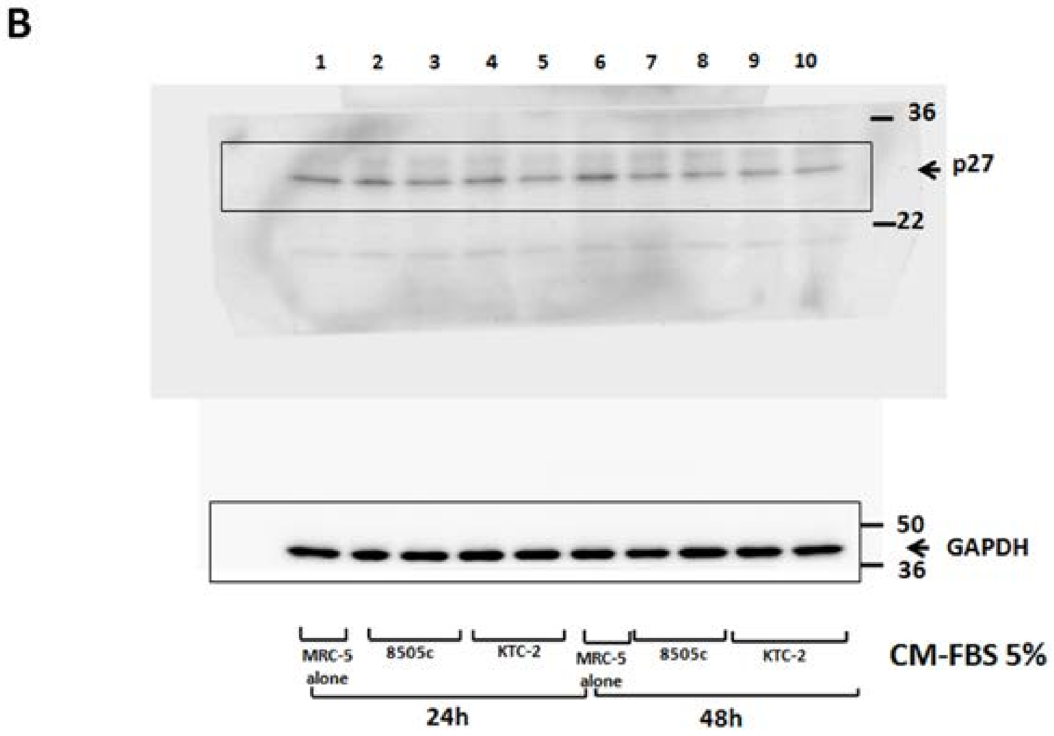

Full Original Blots-III.

Panel C: This figure displays the full original blots for Figure 3G shown in the text/Results. The identification of pSrc bands was based on the expected molecular weight. The same blot was stripped and re-blotted using anti-Total Src antibody.

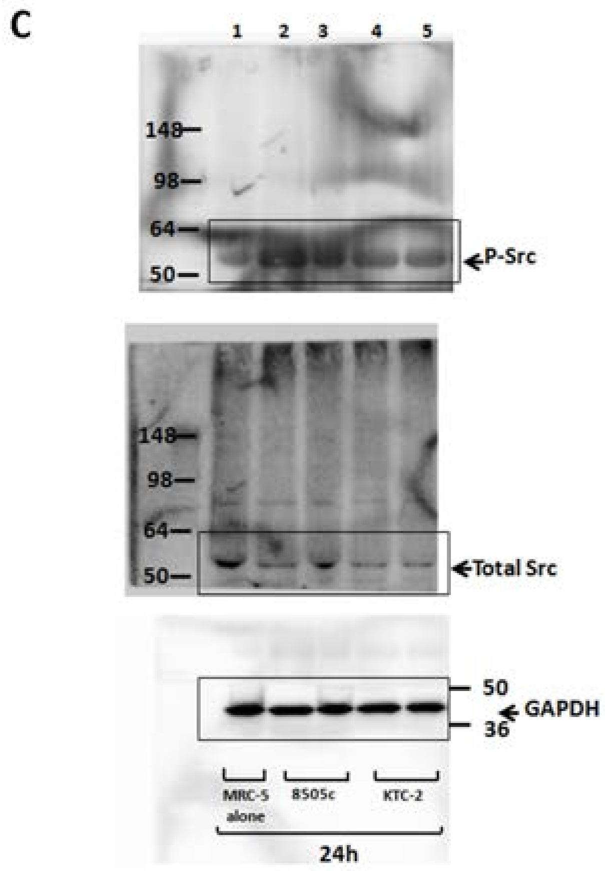

Full Original Blots-III.

Panel D: This figure displays the full original blots for Figure 3I shown in the text/Results. The identification of pAkt bands was based on the expected molecular weight. The same blot was stripped and re-blotted using anti-Total Akt antibody.

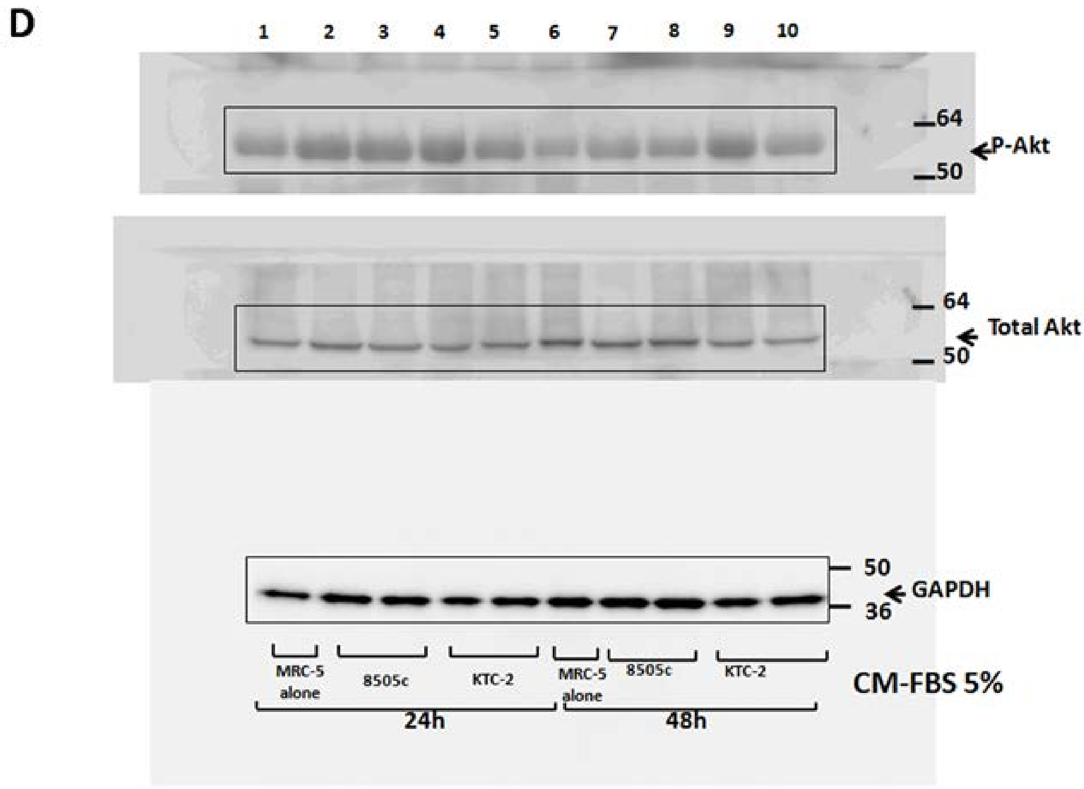

Full Original Blots-IV.

This figure displays the full-length blots for Figure 4A shown in the text/Results.

The identification of Vimentin bands was based on the expected molecular weight. The same blot was stripped and re-blotted using anti-GAPDH antibody for loading control.

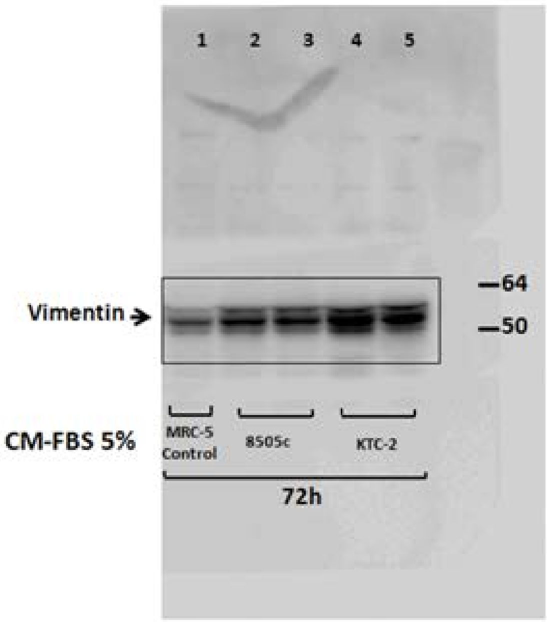

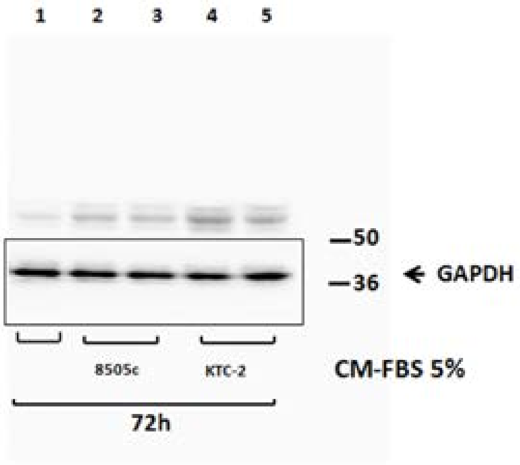

Full Original Blots-VI.

Panel A: This figure displays the full original blots for Figure 6A shown in the text/Results. The identification of PDGFR-β bands was based on the expected molecular weight. The same blot was stripped and re-blotted using anti-GAPDH antibody for loading control.

Panel B: This figure displays the full original blots for Figure 6C shown in the text/Results. The identification of α-SMA bands was based on the expected molecular weight. The same blot was stripped and re-blotted using anti-GAPDH antibody for loading control.

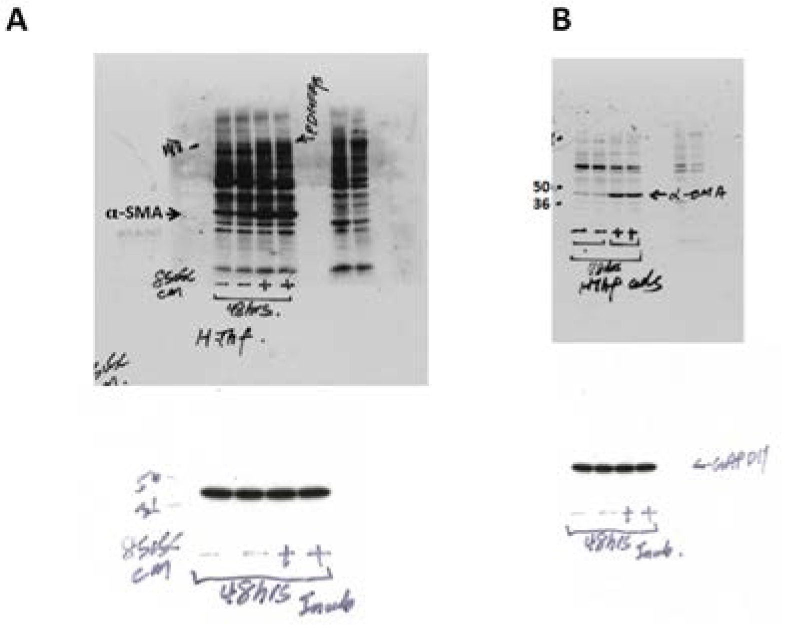

Full Original Blots-VI.

Panel C: This figure displays the full original blots for Figure 6E shown in the text/Results. The identification of Vimentin bands was based on the expected molecular weight. The same blot was stripped and re-blotted using anti-GAPDH antibody.

Panel D: This figure displays the full original blots for Figure 6G shown in the text/Results. The identification of GLUT-1 bands was based on the expected molecular weight. The same blot was stripped and re-blotted using anti-GAPDH antibody.

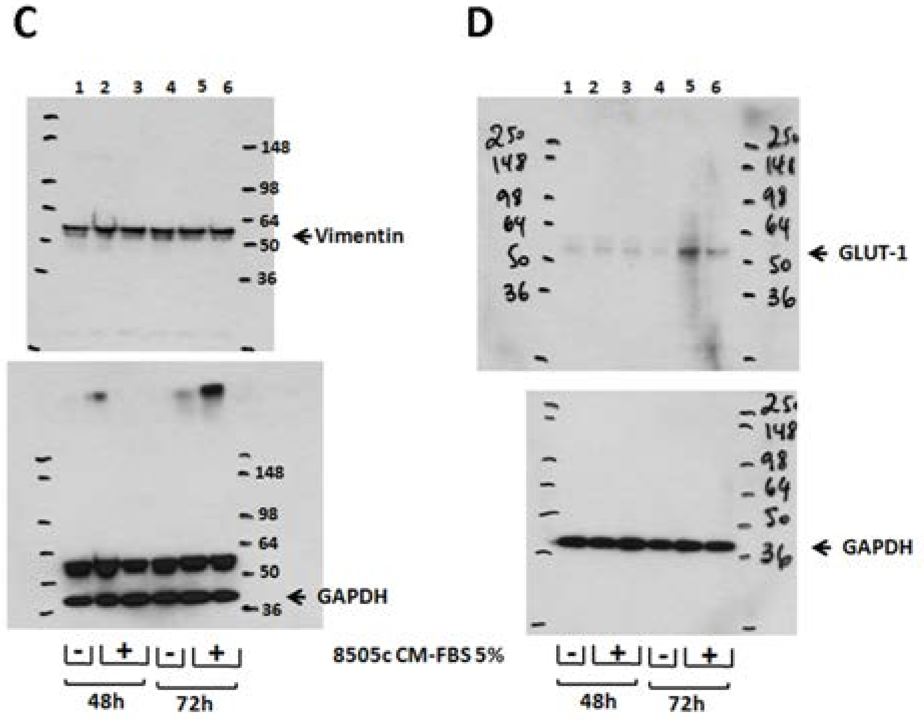

Full Original Blots-VII.

Panel A: This figure displays the full original blots for Figure 7D shown in the text/Results.

The identification of Vimentin bands was based on the expected molecular weight.

The same blot was stripped and re-blotted using anti-GAPDH antibody for loading control.

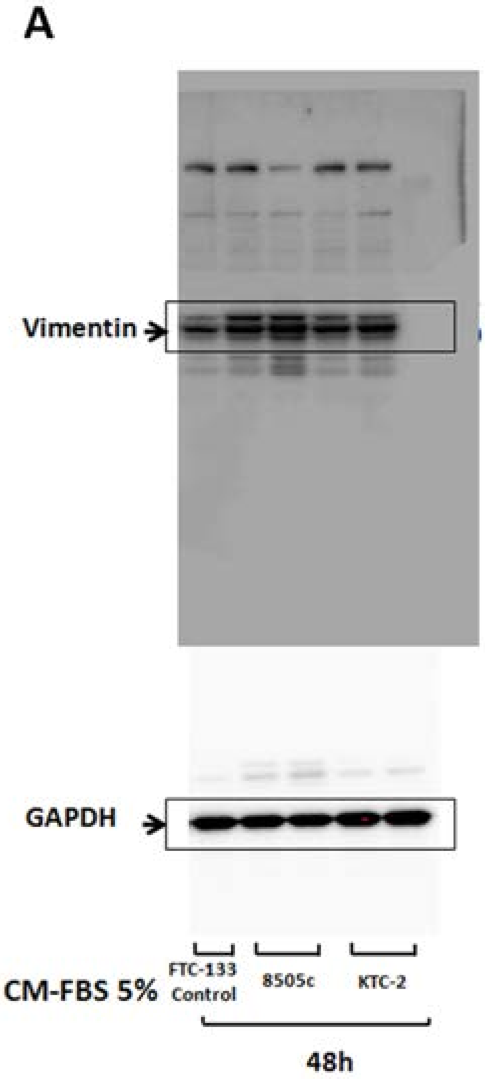

Full Original Blots-VII.

Panel B: This figure displays the full original blots for Figure 7D shown in the text/Results. The identification of E-Cadherin bands was based on the expected molecular weight. The same blot was cut and blotted using anti-GAPDH antibody for loading control.

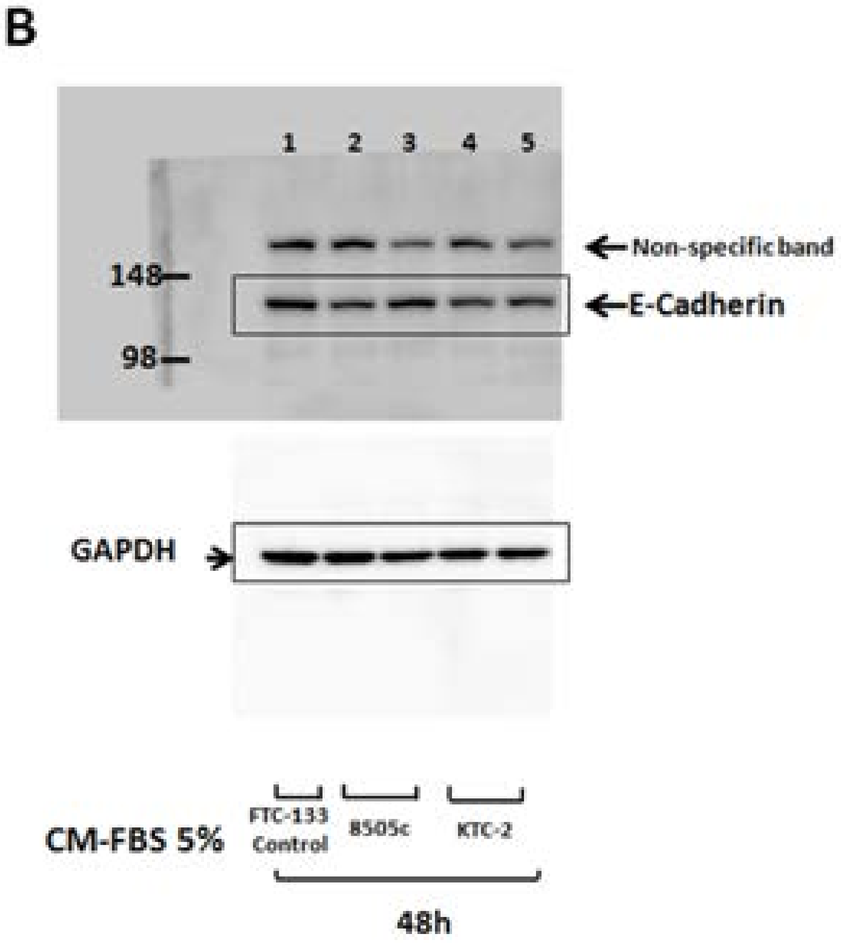

Supplement: Supplementary file 1 — Supplementary Information [file 41598_2019_44361_MOESM1_ESM.pdf]
